# Metabolic regulation of myeloid-derived suppressor cells in tumor immune microenvironment: targets and therapeutic strategies

**DOI:** 10.7150/thno.105276

**Published:** 2025-01-13

**Authors:** Hong Wang, Fei Zhou, Wenqing Qin, Yun Yang, Xiaojiaoyang Li, Runping Liu

**Affiliations:** 1 School of Life Sciences, Beijing University of Chinese Medicine, 11 Bei San Huan Dong Lu, Beijing, 100029, China.; 2 Department of Cancer Biology, Lerner Research Institute, Cleveland Clinic, Cleveland, OH 44195, USA.; 3 School of Chinese Materia Medica, Beijing University of Chinese Medicine, 11 Bei San Huan Dong Lu, Beijing, 100029, China.

**Keywords:** MDSC, TME, metabolism, cancer, immunotherapy

## Abstract

Cancer remains a major challenge to global public health, with rising incidence and high mortality rates. The tumor microenvironment (TME) is a complex system of immune cells, fibroblasts, extracellular matrix (ECM), and blood vessels that form a space conducive to cancer cell proliferation. Myeloid-derived suppressor cells (MDSCs) are abundant in tumors, and they drive immunosuppression through metabolic reprogramming in the TME. This review describes how metabolic pathways such as glucose metabolism, lipid metabolism, amino acid metabolism, and adenosine metabolism have a significant impact on the function of MDSCs by regulating their immunosuppressive activity and promoting their survival and expansion in tumors. The review also explores key metabolic targets in MDSCs and strategies to modulate MDSC metabolism to improve the tumor immune microenvironment and enhance anti-tumor immune responses. Understanding these pathways can provide insight into potential therapeutic targets for modulating MDSC activity and improving outcomes of cancer immunotherapies.

## Introduction

Cancer remains a significant challenge to global public health, with rising incidence and high mortality rates 1. While surgery, radiotherapy and chemotherapy have been the mainstays of treatment, there effectiveness is often limited by severe side effects and inconsistent patient responses 2. Targeted therapies that block specific signaling pathways critical for cancer cell survival have improved outcomes for certain cancers, with notable examples such as inhibitors of epidermal growth factor receptor, HER2, estrogen receptor, and vascular endothelial growth factor receptor, as well as multikinase inhibitors, 3. However, issues such as acquired drug resistance and poor efficacy in certain pathophysiologically complex cancer types continue to impede research progress. Recently, cancer immunotherapies, especially immune checkpoint inhibitors such as anti-PD-1 and anti-PD-L1 antibodies, have made significant advances and even won the 2018 Nobel Prize for the discovery of the mechanism of immune evasion in PD-L1/PD-1 cancers 4, 5. Nonetheless, these therapies benefit only a subset of patients, primarily due to the complexity and heterogeneity of the tumor microenvironment (TME).

TME often referred to as the “devil's sanctuary”, includes immune infiltration, fibroblasts, extracellular matrix (ECM), and blood vessels, creating a space for cancer cell proliferation with minimal inhibition of cancer cells. T cells, dendritic cells (DCs), natural killer (NK) cells, macrophages, Myeloid-derived suppressor cells (MDSCs) and other immune cells are important components. Effector cells such as cytotoxic T lymphocytes (CTLs) and NK cells play a key role in immune surveillance 6. Current immunotherapies, including CAR T cells and checkpoint inhibitors, are designed to enhance the activity of CTLs 7. However, many tumors lack adequate adaptive immune responses (“cold tumors”) due to the presence of immunosuppressive infiltrates such as MDSCs, tumor-associated macrophages (TAMs), and Tregs, thus complicating therapeutic efforts 8.

**Figure 1:**
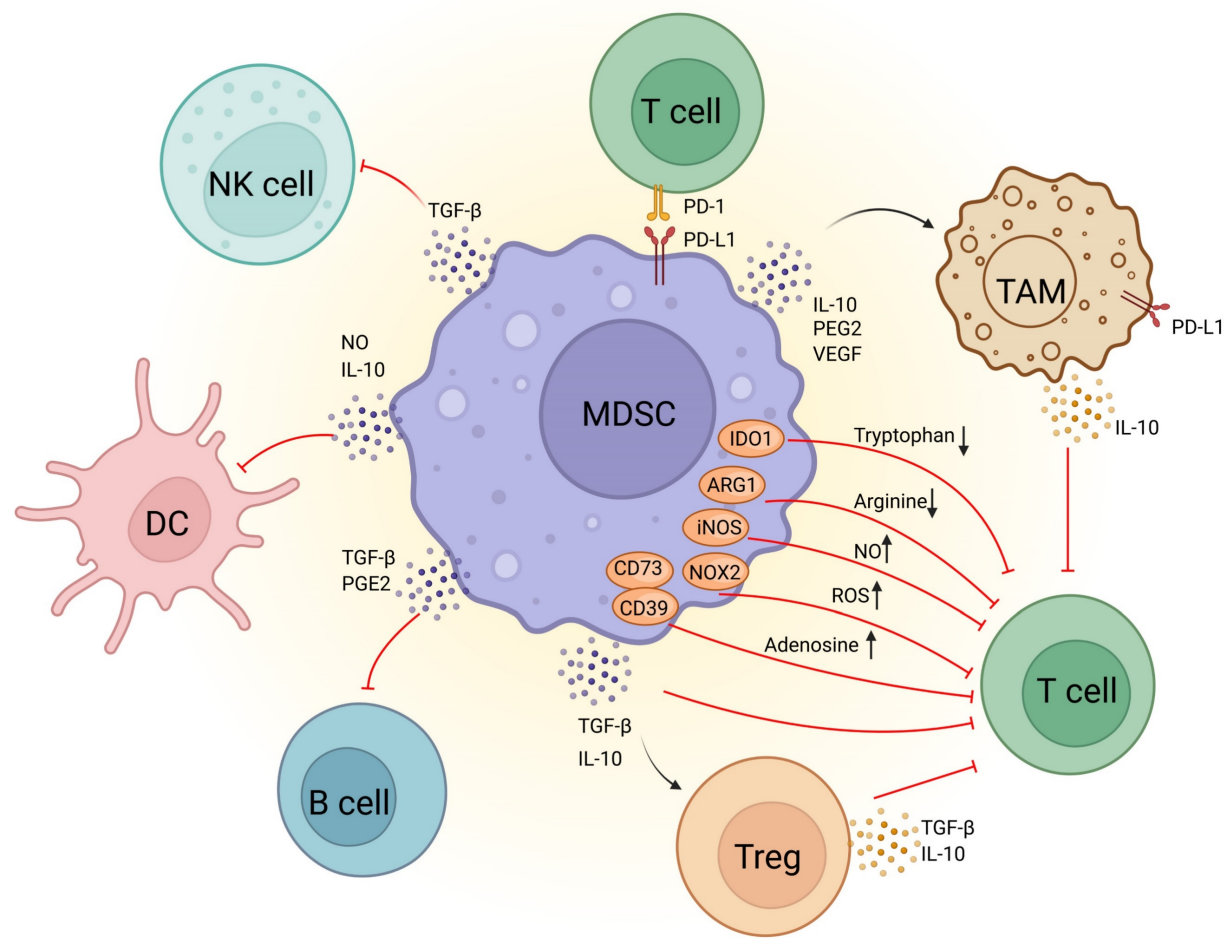
** MDSC in immunodepression in TME.** The molecular mechanisms underlying MDSCs-mediated immunosuppression in the tumor microenvironment. MDSCs suppress T-cell activity through the depletion of essential nutrients such as tryptophan and arginine, as well as by producing high levels of ROS, NO, adenosine, and immunosuppressive cytokines like TGF-β and IL-10. Additionally, MDSCs promote the expansion of Tregs through the secretion of TGF-β and IL-10, which further dampen T-cell function. MDSCs also secrete IL-10, PGE2, and VEGF, which contributes to macrophage polarization. MDSCs express PD-L1, which binds to the PD-1 receptor on T cells, inducing T-cell exhaustion and dysfunction. In addition, MDSCs inhibit B-cell proliferation via TGF-β and PGE2, suppress natural killer (NK) cell function through TGF-β, and impair dendritic cell antigen presentation through the secretion of IL-10 and NO. ARG1, arginase 1, DC,dendritic cell, IDO1, indoleamine 2,3-dioxygenase-1, IL-10, Interleukin 10, iNOS, inducible nitric oxide synthase, MDSC, Myeloid-derived suppressor cell, NK cell, natural killer cell, NO, nitrous oxide, NOX2, NADPH oxidase 2, PD-1, programmed cell death protein 1, PD-L1, programmed death ligand 1, PGE2, prostaglandin E2, ROS, reactive oxygen species, TAM, tumor-associated macrophage, TGF-β, transforming growth factor beta, Treg, regulatory T cell, VEGF, vascular endothelial growth factor.

Among these cells, MDSCs are one of the most abundant myeloid cells within the TME and are thought to be key drivers of immunosuppression (**Figure 1**). Originating in the bone marrow, MDSCs migrate to the tumor site and expand in response to tumor-derived factors and inflammatory signals 9. This expansion is associated with their metabolic reprogramming, including increased glycolysis and lipid metabolism that support their immunosuppressive functions. MDSCs suppress the immune response by inhibiting CTLs activation, promoting the expansion of Tregs, and secreting immunosuppressive cytokines like IL-10 and TGF-β 10. They also increase oxidative stress in TME, impairing CTLs function 11. Furthermore, MDSCs promote tumor progression by enhancing angiogenesis, tumor invasion, metastasis, and drug resistance 12. Thus, targeting MDSCs rather than focusing solely on enhancing CTL efficacy could stimulate a more comprehensive anti-tumor immune response, representing a promising direction for future cancer immunotherapy.

In the current review, we aim to describe how the metabolic state of MDSCs affects their immunosuppressive capacity, identify key metabolic targets of MDSCs in TME, and summarize strategies for targeting MDSC metabolism. In addition, we highlight the advantages of improving the tumor immune microenvironment by inhibiting metabolic regulation of MDSCs, providing insights into improving anti-tumor immune responses by modulating these pathways.

## MDSCs in immunosuppressive TME

The concept of MDSCs were firstly introduced 15 years ago by Gabrilovich, and was familiar to the scientific public as increasing researches on MDSCs have been published in recent years. MDSCs are now considered to be the “queen bee” of the immunosuppressive TME. They inhibit the growth and function of NK cells and CTLs through the release of a variety of immunosuppressive cytokines, which continually trigger a vicious cycle of chronic inflammation in the body.

MDSCs originate during bone marrow hematopoiesis, and their differentiation process is notably complex. Hematopoietic stem cells in the bone marrow differentiate into common myeloid progenitor cells, which then differentiate into granulocyte-monocyte progenitors and monocyte-dendritic cell progenitors. Under normal physiological conditions, monocyte-dendritic cell progenitors next differentiate into monocytes and dendritic cells. In contrast, under pathological conditions, such as tumors, they transform into immunosuppressive mononuclear MDSCs (M-MDSCs) and myeloid cells into polymorphonuclear MDSCs (PMN-MDSCs), which are also referred to as two major subpopulations of MDSCs. Intriguingly, it has been demonstrated that PMN-MDSCs are less immunosuppressive than M-MDSCs. In mice, MDSCs generally express myeloid lineage differentiation markers like Gr-1 and CD11b are defined as MDSCs (CD11b^+^Gr-1^+^), which are further identified into PMN-MDSCs (CD11b^+^Ly6G^+^Ly6C^lo^) and M-MDSCs (CD11b^+^Ly6G^-^Ly6C^hi^) by the expression of Ly6C. However, the flow cytometry results of human are totally different from those in mice, by which human PMN-MDSCs were well characterized as CD11b^+^CD14^-^CD15^+^(or CD66b^+^) and M-MDSCs as CD11b^+^CD14^+^CD15^-^HLA^-^DR^low/-^.

In the bone marrow, pro-cancer and pro-chronic inflammatory factors in TME have been proved to promote the expansion and differentiation of immature myeloid cell into MDSCs, including granulocyte monocyte colony stimulating factor (GM-CSF), granulocyte colony-stimulating factor (G-CSF), macrophage colony-stimulating factor (M-CSF), S100 proteins, interleukin (IL)-6 and tumor necrosis factorα-alpha (TNF-α). After that, the recruitment and expansion of MDSCs to the tumor sites are regulated by multiple tumor-derived factors secreted by TME. These factors can be classified into two distinct types of signals, the first is trafficking signals mainly secreted by tumor cells for recruiting MDSCs into tumors, and the second is activation signals of MDSCs mainly secreted by tumor stroma 13. Chemokines have been proved to play an essential role in the process of recruitment 14, including CCL2/CCL12-CCR2, CCL3/4/5-CCR5, CXCL5/2/1-CXCR2, and CXCL13-CXCR5. In addition, some chemokines have been found not only promote their migration, but also induce the proliferation of MDSCs and maintain their immunosuppressive activity. For instance, CCR5 ligands (CCL3, CCL4, CCL5) have been shown to induce the proliferation of CCR5-expressing MDSCs in the bone marrow, while CCL5 deficiency adversely modulated the morphology, differentiation, and immunosuppressive activity of MDSCs, ultimately inhibiting tumor growth 15. Moreover, recent studies have pointed out that hypoxia at the primary tumor site is linked to the recruitment of CX3CR1-expressing MDSCs, mediated by the activation of CCL26 in cancer cells 16.

Recent studies have demonstrated that the immunosuppressive capacity of MDSCs is influenced by their intracellular metabolic pathways. Representative signals that stimulate MDSCs to acquire immunosuppressive properties include signal transducer and activator of transcription (STAT3), prostaglandin E2 (PGE2) and other significant regulators that are related to the metabolism of both glucose and lipids. MDSCs have also been proved to induce anergy in CD4^+^, CD8^+^ T cells and NK cells through metabolic-based mechanisms that consume essential amino acids. High expression of arginase 1 (ARG1) and inducible nitric oxide synthase (iNOS) in MDSCs is responsible for the depletion of arginine and the production of nitric oxide (NO). Meanwhile, increased expression of NOX2 in MDSCs can produce high level reactive oxygen species (ROS), which can react with NO to form peroxynitrite, abrogating the migration and antigen-specific response in CD8^+^ T cells and CTLs 17, 18 Intriguingly, it has also been found that PMN-MDSCs and M-MDSCs adopt different mechanisms on inhibiting the immune system by regulating metabolic homeostasis in TME. PMN-MDSCs suppress antigen-specific CD8^+^ T cells primarily by the production of ROS, while M-MDSCs inhibit T-cell responses under antigen-specific and nonspecific manners through the expression of the enzymes ARG1 and iNOS as well as the production of ROS. Collectively, metabolic reprogramming of MDSCs in TME regulates their immunosuppressive mechanisms. In the following, we will delve into the metabolic effects on MDSCs' functions from four aspects: glucose metabolism, lipid metabolism, amino acid metabolism, and adenosine metabolism, as well as exploring the possibility of targeting these metabolic pathways as potential drug targets.

## Glucose metabolism

### Glycolysis

**Figure 2:**
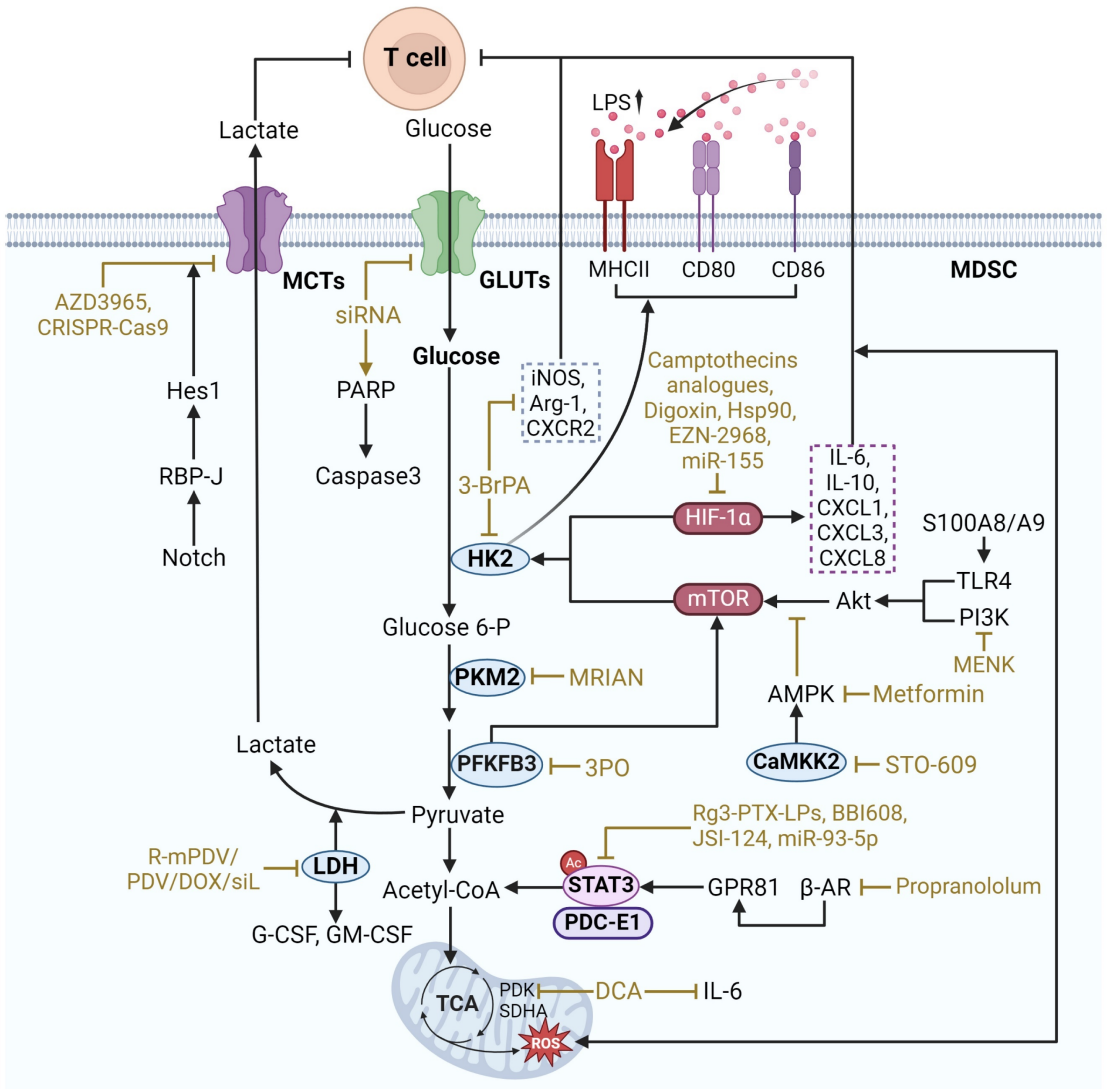
** Therapeutic strategies targeting glucose metabolism in MDSCs.** Acetyl-CoA acetyl coenzyme A, Akt protein kinase B, ARG1 arginase 1, β-AR beta-adrenergic receptor, CaMKK2 calcium/calmodulin-dependent protein kinase kinase 2, Glucose 6-P glucose 6-phosphate, GLUTs glucose transporters, GPR81 G-protein coupled receptor 81, Hes1 hairy and enhancer of split 1, HIF-1α hypoxia-inducible factor 1-alpha, HK2 Hexokinase 2, iNOS inducible nitric oxide synthase, LDH Lactate Dehydrogenase, LPS Lipopolysaccharide, MCTs monocarboxylate transporters, MHCII Major Histocompatibility Complex class II, MPKA mitogen-activated protein kinase, mTOR mammalian target of rapamycin, PAPR poly ADP-ribose polymerase, PDK pyruvate dehydrogenase kinase, PFKFB3 6-Phosphofructo-2-kinase/fructose-2,6-biphosphatase 3, PI3K phosphatidylinositol 3-kinase, PKM2 pyruvate kinase M2, PPP pentose phosphate pathway, RBP-J recombination signal bindingprotein for immunoglobulin kappa J regions, SDHA succinate dehydrogenase flavoprotein subunit, TCA tricarboxylic acid cycle, TLR4 toll-like receptor 4. The black arrows show endogenous changes and the yellow arrows represent effects of potential therapies.

Glycolysis converts glucose in the cytoplasm to pyruvate, resulting in the production of triphosphate (ATP) and NADH, a process that is critical for the function of MDSCs. In a hypoxic TME, glycolysis is better able to provide energy and protection to MDSCs, enabling them to survive and maintain immunosuppressive functions. Enhanced glycolysis contributes to the suppressive effects of MDSCs on effector T cells, thereby facilitating tumor immune escape (**Figure 2**).

The key regulatory targets of glycolysis in MDSCs include multiple types of components as follows. Representative regulators include hypoxia-inducible factor 1-α (HIF-1α), mechanistic target of rapamycin (mTOR), and STAT3. These regulatory factors increase the expression of glucose transporter type 1 (GLUT1) and glucose transporter type 3 (GLUT3), which promotes the uptake of glucose into the cell. Subsequently, glucose entering the cell is phosphorylated by hexokinase 2 (HK2) to initiate glycolysis. Another key enzyme, pyruvate kinase M2 (PKM2), converts phosphoenolpyruvate to pyruvate to catalyze the subsequent process of glycolysis. In addition, pyruvate is also critical for maintaining glycolysis levels. Under anaerobic conditions, pyruvate can be converted to lactate by lactate dehydrogenase A (LDHA). And lactate and pyruvate can be transported across cell membranes by monocarboxylate transporters (MCT). Notably, a broader regulatory role is mediated by calcium/calmodulin-dependent protein kinase kinase 2 (CAMKK2), which affects the overall metabolic response.

### HIF-1α

HIF-1α, an important driver of glycolysis under the hypoxic conditions as well as the downstream factor of PI3K-AKT-mTOR pathway, has been identified to result in increased glucose levels and upregulated glycolytic enzymes and lactate transporters, thus promotes the transition of oxidative phosphorylation (OXPHOS) to glycolysis 19. It's worth noting that upregulation of HIF-1α under hypoxia can enhance the suppressive function of MDSCs by directly upregulating the transcription of PD-L1 on MDSCs, while increased PD-L1 can inhibit the activation of T cells. Through chromatin immunoprecipitation and luciferase reporter experiments, Muhammad *et al.* have revealed that HIF-1α can bind to the transcriptionally active hypoxia-response element in the promoter of PD-L1. Moreover, inhibition of PD-L1 in *ex vivo* MDSCs by an anti-PD-L1 monoclonal antibody significantly decreased the expression of protumor cytokines including IL-6 and IL-10^20^, ^21^. Wang's group also found that HIF-1α can promote the recruitment of MDSCs under the regulation of miR-155. In miR-155 deficient MDSCs, HIF-1α expression was markedly increased and therefore the expression of CXCL1, CXCL3 and CXCL8 was subsequently upregulated, contributing to the accumulation of MDSCs in TME. The research also showed that both Lewis lung carcinoma and B16-F10 melanoma tumors grow faster in miR-155 knockout (miR-155^-/-^) mice, accompanied with a notable increase accumulation of MDSCs in tumors compared with the wild-type mice 22. In addition, Natascha and colleagues have also identified that a reduced number of MDSCs in mice in myeloid HIF-1α knockout mice 23.

Therapies targeting HIF-1α can be mainly divided into 3 subcategories: agents inhibiting HIF-1α transcription, agents inhibiting HIF-1α translation, and agents destructing HIF-1α stabilization. Camptothecins analogues, one of the most widely used topoisomerase I inhibitors, have been proved to suppress HIF-1α protein translation in hypoxic U251 human glioma cells. And the FDA approved camptothecin derivatives including Irinotecan and Topotecan, have been identified with potent antitumor activities against solid tumors. Digoxin, a cardiac glycoside, also inhibits HIF-1α protein expression and a clinical phase 2 trail (NCT01763931) using digoxin on human breast cancer have achieved promising results. EZN-2968 belongs to an RNA antagonist that can binds to the HIF-1α mRNA to inhibit its translation. A clinical study (NCT01120288) of EZN-2968 on duodenal neuroendocrine tumor patients at a dose of 18 mg/kg once a week, observed a significant reduction in HIF-1α mRNA levels 24. Hsp90 inhibitors can block the binding of HIF-1α with Hsp90, which further destabilize HIF-1α. Geldanamycin and its analogs are specific Hsp90 inhibitors. A clinical research (CAUY922A2101) of AUY922 (Geldanamycin derivative) on Japanese patients with advanced solid tumors achieved potential clinical activities 25. Although the regulative effect of most HIF-1a inhibitors on immunosuppressive TME was not determined in clinical and pre-clinical studies, a recent study suggested that these anti-tumor effects of digoxin are at least partially attributed to the decreased recruitment of MDSCs into the TME 16.

### mTOR

mTOR is a major regulator of glycolytic reprogramming in MDSCs, which consists of two complexes: mTORC1 and mTORC2. mTORC1 mainly regulates cell growth and metabolism, whereas mTORC2 is associated with cell survival and cytoskeleton organization. It was found that CXCL1/CXCR2 recruits PMN-MDSCs, and S100A8/A9 increases CXCL1 expression in gastric cancer (GC) through the TLR4/p38 MAPK/NF-κB pathway. Subsequently, PMN-MDSCs aggregated, leading to CD8^+^ T cell exhaustion *via* the S100A8/A9-TLR4/AKT/mTOR pathway, which reduced GC growth and enhanced the efficacy of anti-PD-1 therapy 26. Additionally, mTOR signaling has been shown to be critical for MDSC differentiation. It has been shown that MDSCs in tumors display higher rates of glycolysis and higher inhibitory activity compared to MDSCs in spleen. However, the phosphorylation-dependent activation of key signaling pathways including Akt and MAPK was significantly reduced, and in contrast, mTOR phosphorylation was upregulated in intratumoral MDSCs. Inhibition of mTOR phosphorylation reduced the immunosuppressive activity of intratumoral MDSCs and limited tumor growth by decreasing the level of glycolysis through selective degradation of HK2 21. Seahorse metabolic analyses corroborated that Lipopolysaccharide (LPS) induction significantly enhanced the glycolytic function of MDSCs. And lactate treatment greatly increased the expression of MHCII, CD80 and CD86 in MDSCs to achieve LPS induction and promote the differentiation of MDSCs. During this process, the expression levels of the key glycolytic enzymes PKM2, GLUT1, and PFKFB3 were significantly increased 27. PFKFB3 is a glycolytic enzyme, and PFKFB3-driven glycolysis serves as a feed-forward regulator of mTORC1 signaling to control MDSCs differentiation 28.

Drugs that target mTOR to regulate MDSC glycolysis include Rapamycin, Metformin, Methionine enkephalin, and PFKFB3 inhibitors. Rapamycin is an mTOR inhibitor, which can inhibit mTOR to reduce the expression and activity of glycolytic enzymes. Studies have shown that rapamycin significantly increased the number of MDSCs *in vitro* and inhibited lipopolysaccharide-induced CD80 expression in MDSCs *in vivo*, suggesting that it may affect MDSC differentiation. In addition, rapamycin markedly decreased the expression of iNOS and ARG1 in MDSCs, thereby reducing MDSCs-mediated inhibition of T cell responses 29. Metformin can downregulate the mevalonate pathway by activating AMP-activated protein kinase (AMPK) and inhibiting mTOR, thus reducing the number of MDSCs 30. Methionine enkephalin, on the other hand, can decrease glycolysis and ROS production in MDSCs through the PI3K/Akt/mTOR pathway, thus enhancing immune response 31. Activation of the PI3K-Akt-mTOR pathway significantly upregulated PFKFB3 expression and enhanced aerobic glycolysis 28. PFKFB3 inhibitors, such as 3PO, can minimize mTOR activation by blocking the upstream step of glycolysis, which reduces the glycolytic activity of MDSCs and ultimately improves anti-tumor efficacy 27. These drugs regulate MDSC glycolysis by targeting mTOR and offer potential therapeutic applications.

### STAT3

STAT3, as a transcription factor, plays an essential role in the growth and differentiation of various cells 32. It is also one of the important regulators of glycolytic reprogramming in MDSCs. In TME, lactate accumulation, STAT3 activation, and MDSCs infiltration increase to shape the immunosuppressive microenvironment. The interleukin-6/Janus kinase/signal transducer and activator of transcription 3 (IL-6/JAK/STAT3) inflammatory pathway plays an influential role in limiting effective antitumor immunity in MDSCs 33. Mechanistically, β2-adrenergic receptor (β2-AR) activation triggers STAT3 signaling for metabolic reprogramming through sustained mitochondrial respiration and higher ATP production, improving MDSC function. The STAT3 signaling induced in MDSCs enhances glutamine consumption through the tricarboxylic acid (TCA) cycle. Metabolized glutamine produces itaconic acid, which enhances MDSC survival by downregulating mitochondrial ROS through modulation of Nrf2 and oxidative stress 34. Furthermore, the immunosuppressive microenvironment shaped by MDSCs influences tumor resistance. Radiotherapy enhanced the tumor-promoting activity of MDSCs in pancreatic cancer. The G protein-coupled receptor 81 (GPR81)/mTOR/HIF-1α/STAT3 pathway regulates the Warburg effect enhanced by radiation, causing a sustained increase in lactate secretion. MDSCs stimulated by lactate displayed upregulated expression of tumor-promoting functional genes (S100A8/A9, ARG1, MMPs) and more potent immunosuppressive activity against T cells 35.

Targeting STAT3 to regulate glycolysis in MDSCs involves a variety of strategies. One approach is to directly inhibit STAT3 phosphorylation, thereby decreasing glycolytic enzyme expression and impairing glycolytic flux in MDSCs. For example, STAT3 inhibitor Napabucasin (also known as BBI608), which inhibits the immunosuppressive potential of MDSCs without limiting anti-tumor T-cell responses 36. JSI-124 is a STAT3 inhibitor that inhibits the proliferation of CRC cells. miR-93-5p, an IL-6-driven G-MDSC exosome, promotes the differentiation of M-MDSCs into M2 macrophages and is implicated in the STAT3 signaling mechanism that promotes the transition from colitis to cancer. Combining the STAT3 inhibitor JSI-124 with a strategy to inhibit IL-6-mediated production of the G-MDSC exosome miR-93-5p facilitates the prevention and treatment of colitis-associated cancer 37. Another strategy is to target upstream and downstream effectors of STAT3. The orphan drug dichloroacetate is a small molecule inhibitor of mitochondrial pyruvate dehydrogenase kinase (PDK). PDK inhibition leads to reactivation of pyruvate dehydrogenase in the mitochondria, thereby increasing the ratio of glucose oxidation to glycolysis. Dichloroacetate significantly down-regulates STAT3 activation (phosphorylated STAT3, p-STAT3) and IL-6 expression, thereby down-regulating glycolysis and effectively reducing the immunosuppressive activity of MDSCs. The β-blocker propranololum improves Doxorubicin efficacy by blocking β-AR-induced STAT3 signaling in MDSCs and thus improves survival in EL4 lymphoma model 34. Blocking lactate production in tumor cells or inhibiting the GPR81 receptor, which mediates lactate signaling, disrupts the mTOR/HIF-1α/STAT3 axis, thereby decreasing the glycolytic and immunosuppressive activities of MDSCs 35.

### GLUT1/3

GLUT1 and GLUT3 are glucose transporters that play vital roles in regulating the initial steps of glucose uptake, providing sufficient glucose for glycolysis and subsequent adenosine ATP production 38, 39. Mechanistically, GLUT1 and GLUT3 are known to maintain the high glycolytic flux required to carry out immunosuppressive activities in MDSCs in TME. It was shown that GLUT1 overexpressed in MDSCs transports excess glucose into MDSCs, thereby creating a high-glucose environment within MDSCs. Furthermore, the data suggest that GLUT1 overexpression is a unique feature of MDSCs and that terminal maturation of MDSCs downregulates GLUT1 40. Besides, GLUT1-dependent glycolysis is required for tumor-induced MDSC differentiation and the process is associated with latent membrane protein 1 (LMP1) expression. LMP1 interacts with GLUT1 by disrupting its K48-linked ubiquitination and autolysosomal degradation in a p62-dependent manner and stabilizes the GLUT1 protein by inducing up-regulation of GLUT1 mRNA and protein levels through p65 activation 41. Similarly, GLUT3 is associated with this metabolic adaptation, and inhibition of GLUT3 reduces the inhibitory capacity of MDSCs and enhances anti-tumor immune responses. In 4T1 and 4T07 mouse tumor models, CD205 PMN-MDSCs strongly suppress CD8^+^ T cell antitumor response activity. It was found that CD205 PMN-MDSCs rapidly adapted by increasing GLUT3 expression under TME low glucose stress. *In vitro* and *in vivo* experiments showed that siRNA effectively blocked GLUT3 expression, reduced glucose uptake, and upregulated the caspase3/PARP apoptotic axis in MDSCs cells, especially the CD205 subpopulation. GLUT3 enhanced the resistance of MDSCs to low-glucose stress by competing for glucose and preventing ROS-mediated apoptosis in MDSCs 42.

Targeted inhibition of these transporter proteins reduces glycolysis in MDSCs. Unique Rg3-based liposomes were formulated using ginsenoside Rg3 and loaded with paclitaxel (PTX), namely Rg3-PTX-LPs. Rg3-PTX-LPs could be uptake *via* GLUT1 and specifically distributed in MCF7/T tumor cells and TME. Rg3-PTX-LPs remodeled the immunosuppressive microenvironment by inhibiting the activation of IL-6/STAT3/p-STAT3 pathway, reduced the number of MDSCs, promoted tumor cell apoptosis, and increased the cancer suppression rate 43. Furthermore, it was reported that GSK3β inhibitors may selectively kill cancer cells overexpressing GLUT3 while barely affecting cells expressing only GLUT1.Thus, GLUT3 inhibitors may also exhibit selective elimination of PMN-MDSCs 42, however relevant studies have not been confirmed.

### HK2

HK2 is an enzyme that catalyzes glycolysis by converting glucose to glucose-6-phosphate and facilitating the initial phosphorylation of glucose 44. Mechanistically, HK2 plays an essential role in regulating glycolysis in MDSCs. It has been implicated that the gut fungus Candida tropicalis may enhance the immunosuppressive function of MDSCs by up-regulating HK2. Furthermore, HK2 was found to be part of the Syk-PKM2-HIF-1α axis, which emphasized that HK2 maintains the survival and function of MDSCs in TME by regulating glycolysis levels in MDSCs 45.

Inhibiting HK2 activity is capable of targeting the regulation of glycolytic metabolism in MDSCs. The use of specific inhibitors that block HK2 function with 3-bromopyruvate (3-BrPA) decreases the level of glucose phosphorylation and subsequent glycolysis, thereby reducing the energy supply of MDSCs and their immunosuppressive capacity. It was found that 15 μM 3-BrPA could severely inhibit ATP production in tumor cells by disrupting the interaction between HK2 and mitochondrial voltage-dependent anion channel-1 (VDAC1) proteins, and that glycolysis, NADP, ATP, and lactic acid production were severely inhibited in 40 μM 3-BrPA-treated tumor cells. 15 and 20 mg/kg of 3-BrPA significantly reduced tumor growth in a mouse pancreatic cancer model. The tumors showed apoptotic death with complete inhibition of HK2 and TGFβ, and enhanced expression of active cysteine asparaginase-3. Meanwhile, TME was improved and the number of MDSCs was severely suppressed, which may account for the increased CD8^+^ T cell infiltration and tumor growth inhibition 46. In conclusion 3-BrPA showed good effects on metabolic regulation of tumor cells, thus exerting anti-tumor effects. Therefore, whether 3-BrPA could directly regulate the metabolic reprogramming of MDSCs and thus affect their differentiation and function? An *in vitro* study found that 3-BrPA significantly inhibited the glycolysis of MDSCs, increased the expression of MDSCs-related immunosuppressive molecules, such as iNOS, ARG1, and CXCR2, enhanced the expression of PD-L1, and promoted the differentiation of the CD155 phenotype of MDSCs, which can inhibit the function of T-cells and effector cells 47. However the correlation between metabolic reprogramming and immune function in this result is contradictory to other studies and may be caused by the fact that this is an *in vitro* study, which is divorced from the interaction of immune cells in the TME.

### PKM2

PKM2, a member of pyruvate kinase, also functions as a pivotal enzyme for glycolysis. Activation of PKM2 is closely related to the proliferation, invasion, and metastasis of cancer cells 48. A recent study on 721 HCC patients revealed not only an oncogenic role for PKM2 but also an effective function for recruiting MDSCs to the tumor site. By transplanting PKM2-knockdown HCCLM3 cells into mice, it was found that tumor progression was significantly inhibited and the percentage of CD11b^+^Gr-1^+^ granulocytic MDSCs and CD11b^+^Ly6C^+^ monocytic MDSCs were decreased obviously when compared with HCCLM3 transplanted mice. Mechanistically, the knockdown of PKM2 in HCCLM3 cells increased CXCL1 and MIF, which function as important cytokines to recruit MDSCs 49.

In T cell acute lymphoblastic leukemia therapy, metabolically reprogrammed immunosurveillance-activated nanomedicine (MRIAN), a novel amino acid metabolite nanomedicine, was applied. MRIAN was capable of degrading to L-phenylalanine, inhibiting PKM2 activity and decreasing ROS levels in MDSCs, thereby interfering with their immunosuppressive function 50. While, in the treatment of colorectal cancer, a mesoporous polydopamine nanoparticle (SHK@HA-MPDA) utilizing alizarin-loaded, hyaluronic acid-modified nanoparticles is effective in the treatment of colorectal cancer liver metastases (CRLM). SHK@HA-MPDA provides an approach against CRLM by targeting both metabolic and immune pathways by inhibiting PKM2 and glycolysis 51. Targeting the Syk-PKM2-HIF-1α axis effectively reduces glycolysis in MDSCs. Studies have shown that inhibition of PKM2 or blocking its nuclear translocation attenuates the enhancement of MDSC glycolytic activity induced by intestinal fungi. In Candida tropicalis-treated MDSCs, PKM2 Tyr105 phosphorylation and PKM2 nuclear translocation were required and increased the interaction between PKM2 and HIF-1α. MDSCs in CRC showed elevated expression of PKM2, PKM2 (p-Y105), and iNOS, with positive correlation with MDSCs infiltration. siPKM2 markedly blocked the mRNA and protein expression of HIF-1α-dependent glycolytic enzymes in Candida tropicalis-treated MDSCs. Meanwhile, PKM2 knockdown also decreased the mRNA and protein expression of iNOS, Cyclooxygenase 2 (COX2) and NADPH oxidase 2 in Candida tropicalis-stimulated MDSCs. In conclusion, PKM2 Tyr105 phosphorylation and PKM2 nuclear translocation are essential for HIF-1α-dependent glycolytic metabolism in Candida tropicalis-induced MDSCs 45.

### LDH

LDH is an enzyme that catalyzes the conversion of pyruvate to lactate during glycolysis, playing a crucial role in anaerobic metabolism 52. It allows the regeneration of NAD^+^, enabling the continuation of glycolysis under anaerobic conditions 53. LDHA is one of the isoforms of LDH responsible for converting pyruvate to lactate during glycolysis 54. Mechanistically, LDHA expression is upregulated in MDSCs to promote lactate production, and elevated lactate levels further activate HIF-1α to promote glycolytic enzyme expression and enhance glycolytic flux in MDSCs 55. LDH-A-produced lactate activates the glycolytic pathway, upregulates CD4^+^ T cell proliferation, and increases the release of immunosuppressive effector molecules, such as ARG1 and iNOS, from MDSCs 56. Moreover, LDHA is usually accompanied by abundant tumor-derived G-CSF and GM-CSF, which further promotes the recruitment and immunosuppression of MDSCs 57.

LDHA knockdown may affect MDSC recruitment through autophagy-related mechanisms. To achieve potent anti-tumor immunity, a redox-responsive nano-assembly (R-mPDV/PDV/DOX/siL)-based immunochemotherapy regimen was developed, which integrates a combined strategy of inhibiting cytokine-mediated MDSC recruitment through LDHA silencing and enhancing tumor immunogenicity through anthracycline (DOX)-induced immunogenic cell death effects. After R-mPDV/PDV/siL treatment, LDHA mRNA downregulation by more than 80% and lactate accumulation were significantly reduced in 4T1 tumors, along with activation of AMPK-ULK1 pathway and autophagy in tumor cells, and reduction of G-CSF and GM-CSF production. Meanwhile, R-mPDV/PDV/siL also reduced the infiltration of MDSCs, lowered the level of immunosuppressive IL-10, and increased the ratio of CD4^+^ T cells and CD8^+^ T cells in tumors 57. Moreover, the use of pd-1 antibodies has a modulating effect on LDH. Melanoma patients have elevated levels of total MDSCs after receiving anti-PD-1 immunotherapy (navulizumab and pabolizumab). Among them, LDH levels were correlated with poor prognosis of anti-PD-1 therapy, and serum LDH levels were significantly reduced in those who responded to checkpoint therapy, suggesting the therapeutic promise of our inhibition of LDH in combination with PD-1 antibodies 55. Thus, targeting LDH in MDSCs to modulate metabolism focuses on the potential of inhibiting LDHA to disrupt glycolysis and reduce lactate production as a means to modulate MDSCs activity and improve cancer treatment efficacy.

### MCT

MCTs are proteins that facilitate the transport of lactate, pyruvate, and other monocarboxylates across cell membranes and play a critical role in cellular metabolism and pH regulation 58. Pyruvate, the end product of glycolysis, which can be reduced to lactate in the cytosol. After transported by MCT, the lactate that aggregates in the TME shapes the acidic microenvironment of the TME and supports the metabolism of immunosuppressive cells such as MDSCs and Treg 59, 60. Lactate can also act as a signaling molecule enhancing the immunosuppressive phenotype of MDSCs by stabilizing HIF-1α and upregulating glycolytic enzymes 61, 62.

Disrupting lactate output by inhibiting these transporters can impair glycolytic metabolism in MDSCs. Specific inhibitors of MCTs, such as AZD3965, have been shown to diminish lactate transport, result in intracellular lactate accumulation, reduce glycolytic flux, and decrease immunosuppressive activity in MDSCs 63. Another specific targeting inhibitor of MCTs, AR-C155858 and BAY-8002, have been gradually applied in antitumor research, however current studies have mainly focused on its role in targeting tumors, and the role of targeting MDSCs has not yet been clarified 58. Suppressing the expression of MCTs is another potential approach, since it has been reported that activated Notch/RBP-J signaling represses MCT2 transcription in MDSCs* via* Hes1-binding genes 61. Moreover, knocking down MCTs using CRISPR-Cas9 technology and combining them with immunotherapeutic strategies, such as anti-PD-1 can enhance anti-tumor immune responses and further improve therapeutic efficacy 64.

### CaMKK2

CaMKK2 plays a crucial role in various cellular processes by activating downstream kinases regulated by metabolism, including glycolysis 65. In the context of MDSCs, CaMKK2 regulates glycolysis by activating AMPK. This activation increases the expression of key glycolytic enzymes, which enhances glycolytic flux within MDSCs and supports the immunosuppressive function of MDSCs in TME 66. The most widely used CaMKK2 antagonist is the competitive inhibitor STO-609. STO-609 can reduce glycolysis by downregulating AMPK activation, thereby impairing the immunosuppressive capacity of MDSCs and potentially enhancing anti-tumor immune responses 66.

### Other plausible targets regulating glycolysis in MDSCs

In regulating the glycolytic metabolism of MDSCs, glucose-6-phosphate dehydrogenase (G6PD) plays a crucial role by controlling key steps in the pentose phosphate pathway (PPP), thereby modulating NADPH production and antioxidant protection. Inhibition of G6PD can decrease NADPH production, leading to impaired nucleotide synthesis and cell cycle arrest, which subsequently inhibits tumor cell proliferation and the immunosuppressive functions of MDSCs. Studies have shown that the antifungal drug terbinafine reduces the NADP^+^/NADPH ratio, inhibiting G6PD activity, thereby decreasing MDSC infiltration and tumor burden 67.

Succinate dehydrogenase subunit A (SDHA) is a critical component of TCA and OXPHOS pathway. Modulation of SDHA can significantly impact the energy metabolism and function of MDSCs. Inhibition of silent information regulator 2 can increase NAD^+^ levels, enhance SDHA activity, and elevate OXPHOS levels, thereby augmenting the immunosuppressive activity of MDSCs and improving graft survival rates in transplantation 68. Conversely, blocking SDHA or OXPHOS can significantly restore the immunosuppressive activity and inflammatory cytokine production in SIRT2-deficient MDSCs.

NADH is a vital electron donor in the cellular respiratory chain, participating in energy metabolism and redox reactions. Strategies targeting NADH to regulate MDSC function include the use of hydroxyurea (HU) and its derivatives, such as Mito-HU. These derivatives increase hydrophobicity, inhibit oxidative phosphorylation, and reduce tumor cell proliferation 69. Furthermore, these compounds can effectively inhibit the immunosuppressive function of MDSCs and enhance T cell responses, presenting potential antitumor immunomodulatory effects.

In summary, strategies targeting the regulation of G6PD, SDHA, and NADH can effectively inhibit the immunosuppressive functions of MDSCs and enhance antitumor immune responses by altering their metabolic pathways. These mechanisms and strategies provide new insights and directions for the development of antitumor therapies targeting MDSCs.

## Lipid metabolism

### Fatty acid oxidation

**Figure 3:**
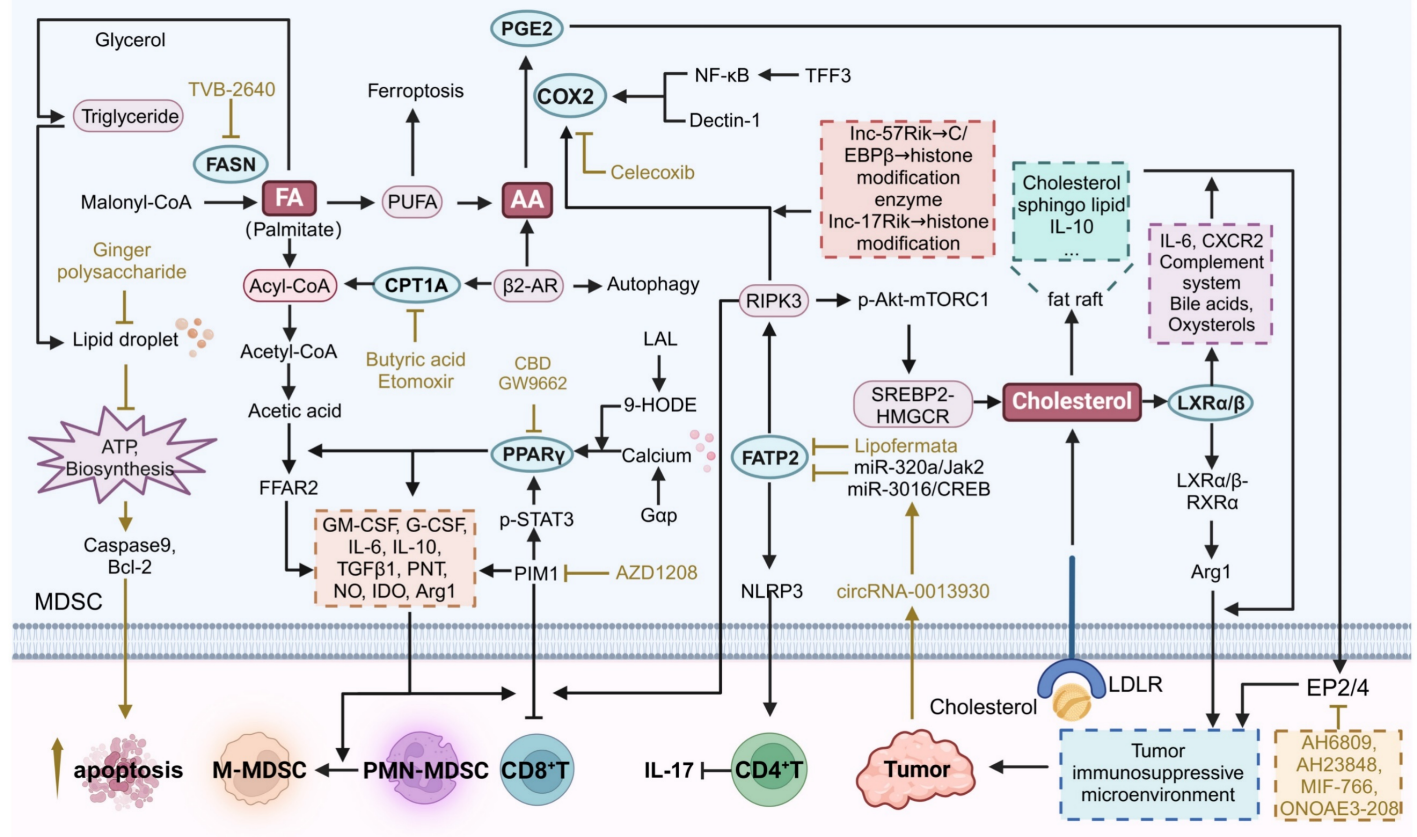
** Therapeutic strategies targeting lipid metabolism in MDSCs.** 9-HODE 9-hydroxyoctadecadienoid acid, AA arachidonic acid, COX2 cyclooxygenase-2, CPT1A carnitine palmitoyltransferase 1A, Gαp guanine nucleotide-binding protein alpha subunit, LDLR low-density lipoprotein receptor, FA fatty acid, FASN fatty acid synthase, FATP2 fatty acid transport protein 2, FFAR2 free fatty acid receptor 2, LAL Lipoarabinomannan, NLRP3 NLR family pyrin domain containing 3, PIM1 proviral integration site for moloney murine leukemia virus 1, PGE2 prostaglandin E2, PUFA polyunsaturated fatty acid. The black arrows show endogenous changes and the yellow arrows represent effects of potential therapies.

Fatty acid oxidation (FAO) is an important metabolic pathway that catabolizes fatty acids primarily in mitochondria and peroxisomes to produce ATP 70. It supports energy homeostasis and is then translocated to mitochondria where it undergoes β-oxidation via the TCA cycle to produce ATP 71. In MDSCs, FAO is essential for their immunosuppressive function and survival within the TME. The enhancement of FAO enables MDSCs to adapt to nutrient-deprived and hypoxic conditions, thereby facilitating their role in suppressing T cell activity and promoting tumor growth (**Figure 3**) 72.

Targeting FAO pathway in MDSCs may provide novel therapeutic strategies to modulate their activity and improve cancer treatment outcomes. The key metabolic targets include peroxisome proliferator-activated receptor γ (PPARγ), carnitine palmitoyltransferase 1A (CPT1A), and stearoyl-CoA desaturase 1 (SCD1), among others. PPARγ regulates genes involved in lipid metabolism, enhances FAO, and supports cellular function 73. CPT1A transports long-chain fatty acids into mitochondria for β-oxidation, which is critical for the development and growth of tumors. 74. These targeting strategies could disrupt FAO in MDSCs, thereby reducing their immunosuppressive activity and enhancing antitumor efficacy.

### PPARγ

PPARγ plays a critical role in regulating FAO in MDSCs. Cannabidiol (CBD) has been shown to induce PPARγ activation in mast cells, leading to G-CSF secretion and subsequent MDSC mobilization 75. In lung adenocarcinoma, the final metabolite of FAO, acetic acid, contributes to immune suppression *via* free fatty acid receptor 2 (FFAR2). This pathway is significantly mediated by the Gαq/calcium/PPARγ/ARG1 axis. FFAR2 deficiency in MDSCs reduces ARG1 expression, relieving L-arginine consumption in the TME, thereby restoring T cell function. Replenishing L-arginine or inhibiting PPARγ with GW9662 can mitigate the acetic acid/FFAR2-mediated suppression of T cells and overcome resistance to immune checkpoint blockade 76. Pharmacological inhibition of PPARγ using a specific inhibitor T0070907 has also been shown to convert the TME from immunosuppressive to immunostimulatory, thereby resensitizing tumors to anti-PD-1 therapies 77. lysosomal acid lipase (LAL) hydrolyzes cholesteryl esters and triglycerides in cytosolic lysosomes to produce free fatty acids and cholesterol, and a downstream metabolic derivative of LAL, 9-hydroxyoctadecadienoic acid (9-HODE), is a hormonal ligand for PPARγ Targeting PPARγ using 9-HODE can regulate the mTOR pathway and subsequently reduce ROS production, which is critical for the suppressive function of MDSCs 78.

Another effective strategy involves the inhibition of PIM kinase, which regulates lipid oxidative metabolism *via* PPARγ activities. A recent study on immunotherapy-resistant tumors using single-cell RNA sequencing revealed that MDSCs with enhanced FAO dominate the immune landscape. In these MDSCs, PIM1 was highly expressed, and PIM1-mediated phosphorylation of STAT3 at the S727 site amplified STAT3's transcriptional activity, leading to increased expression of PPARγ, a key downstream target of PIM1 that promotes FAO and immunosuppression in MDSCs 79. Pharmacologic inhibition of PIM kinase by AZD1208, a potent and selective pan-PIM kinase inhibitor, disrupts the immunosuppressive microenvironment mediated by myeloid cells and enhances CD8^+^ T cell-mediated antitumor immunity, showing potential in overcoming resistance to immune checkpoint blockade therapies 79. This was achieved by targeting PPARγ in MDSCs, reducing FAO, and lowering the expression of indoleamine 2,3-dioxygenase 1 (IDO), ARG1, and TGFβ1 79. These strategies collectively highlight the therapeutic potential of modulating PPARγ to improve cancer immunotherapy outcomes.

### CPT1A

The fatty acid transporter protein CPT1A is required for FAO-mediated immunosuppression of MDSCs. CPT1A is the rate-limiting enzyme of FAO in many tissues that catalyzes the transfer of long-chain acyl group of acyl-CoA ester to carnitine, thereby shuttling long-chain fatty acids into the mitochondrial matrix through the carnitine transporter for β-oxidation 80. Under stress, β2-AR signaling is activated in MDSCs, resulting in increased CPT1A expression. This activation shifts the metabolic balance from glycolysis to oxidative phosphorylation and FAO, and β2-AR signaling induces autophagy and activates the arachidonic acid cycle, which increases the synthesis and release of the immunosuppressive mediator PGE2 and enhances the immunosuppressive capacity of MDSCs 81. The critical role of PGE2 and AA in regulating MDSCs will be further discussed later. The gut microbiota-derived metabolite butyrate was able to activate the CPT1α-dependent FAO pathway to facilitate the acetylation of lysine at position 27 of histone H3 in the promoter region of the PPARD and other FAO genes in MDSCs. By improving impaired mitochondrial function, Butyrate promoted MDSCs amplification and immunosuppressive activity 82.

From a therapeutic perspective, targeting CPT1A and the associated metabolic pathways can modulate MDSCs activities to improve immune responses against tumors. Etomoxir is a specific inhibitor of CPT1. Studies have shown that in normal C57BL/6 mice, treatment with Etomoxir reduces basal and maximal OCR of BM-MDSC, decreases fatty acid uptake, and lowers ATP levels by approximately 40-50%. In addition, Etomoxir treatment significantly reduced the ability of BM-MDSC to block T cell proliferation. In 3LL lung cancer and MCA-38 colon cancer models, Etomoxir treatment reduced the enzymatic activity of CPT1 in T-MDSCs *in vivo* and decreased the overall metabolic activity of T-MDSCs by inhibiting FAO. More importantly, inhibition of FAO* in vivo* decreased the expression of ARG1, ROS, NO, and PNT, which attenuated the immunosuppressive function of T-MDSCs, rendering them unable to block T-cell proliferation and interferon-gamma (IFN-γ) production. Etomoxir treatment led to a significant reduction in the expression levels of cytokines G-CSF, GM-CSF, IL6, and IL10, and subsequently inhibited the induction and differentiation of the immunosuppressive phenotype in MDSCs. However, the high embryonic lethality of CPT1 knockout mice and the fact that conditional CPT1 knockout mice have not been able to be tested have made it impossible to genetically confirm the effects of CPT1 suppression at this time 72.

### Lipogenesis

The lipogenesis pathway, or *de novo* fatty acid synthesis, converts acetyl-CoA to fatty acids in the liver and adipose tissue. Key steps include the carboxylation of acetyl-CoA by acetyl-CoA carboxylase to form malonyl-CoA, which is elongated by fatty acid synthase (FASN) to produce palmitate. Palmitate can be further modified into various fatty acids, esterified with glycerol to form triglycerides, stored in lipid droplets, or used for membrane biosynthesis and energy production. MDSCs require large amounts of energy and biosynthetic precursors during amplification and enhanced functional activity. Through *de novo* fatty acid synthesis, MDSCs can maintain their membrane lipid renewal, energy storage, and signaling molecule synthesis. Fatty acid synthesis, adipogenesis and fat accumulation are associated with the inhibitory function of MDSCs. When fatty acids are excessive within MDSCs, they can be converted to triglycerides, leading to the formation of lipid droplets that promote cell proliferation 83. Thus, MDSCs exhibit a greater reliance on FASN-catalyzed *de novo* fatty acid synthesis than normal cells. Targeting FASN can disrupt these processes and enhance the efficacy of cancer immunotherapy (**Figure 3**).

Inhibition of FASN increases polyunsaturated fatty acids (PUFA), and accumulation of PUFA may lead to iron-dependent cell death 84. So far, except for TVB-2640, no compounds that selectively inhibit FASN have been studied in the clinic. TVB-2640 is a potent and reversible inhibitor of FASN and has been validated in a variety of tumor cell lines, including KRAS-mutant non-small cell lung cancer (NSCLC), colon cancer, advanced HER2 breast cancer, and glioblastoma, as well as in clinical studies 85. Although TVB-2640 was not evaluated for its effects on MDSCs, a study found that a natural compound ginger polysaccharide has similar effects to FASN inhibitors, thereby reducing fatty acid synthesis and lipid droplet formation in MDSCs. This metabolic disruption impairs the energy supply, resulting in elevated levels of pro-apoptotic caspase 9 and reduced levels of anti-apoptotic Bcl-2, thereby promoting the cell death of MDSCs 83.

### Cholesterol metabolism

Cholesterol metabolism encompasses the synthesis, transport, and regulation of cholesterol within the body. It begins with the *de novo* synthesis of cholesterol in the liver from acetyl-CoA, primarily through the mevalonate pathway. Most cells are able to take up cholesterol from low-density lipoproteins (LDL) from the circulation via LDL receptor (LDLR)-mediated nonphagocytosis 86. In TME, cholesterol is not only an important component of cell membranes, but also an important factor in the regulation of immune system functions. It is involved in a variety of immune processes, including lipid raft-mediated signaling and immune cell development and function. Lipid rafts are cholesterol- and sphingolipid-enriched microregions in the cell membrane, providing a platform for the clustering of a variety of signaling molecules such as PD-L1, and, IL-10 in MDSCs 87, 88. These molecules transmit inhibitory signals more efficiently through lipid rafts, thereby promoting the immunosuppressive activity of MDSCs and inhibiting the activation and proliferation of effector T cells (**Figure 3**). Intracellular cholesterol levels are tightly and finely regulated by esterified cholesterol or oxygenated sterols and nuclear receptors such as liver X receptors (LXRs) 89.

When cholesterol synthesis is disrupted, for instance, in receptor-interacting protein kinase 3 (RIPK3)-deficient MDSCs, a significant decrease in cholesterol levels is observed, leading to reduced phosphorylated AKT-mTORC1 signaling and blunted downstream SREBP2-HMGCR-mediated cholesterol synthesis. This cholesterol deficiency, paradoxically, enhances the immunosuppressive activity of MDSCs by promoting the nuclear accumulation of LXRβ. The LXRβ-RXRα heterodimer then binds to a novel composite element in the promoter of ARG1, further augmenting the immunosuppressive properties of MDSCs 90. Further insights into the role of LXRs, particularly LXRα, reveal that systemic or hepatocyte-specific activation of LXRα sensitizes mice to liver tumorigenesis by upregulating the IL-6/STAT3 signaling pathway and the complement system, while downregulating bile acid metabolism. LXRα gain-of-function models, VP-LXRα knockin (LXRαKI) mice and VP-LXRα transgenic mice were used to investigate the role of LXRα activation in liver carcinogenesis. This activation leads to the accumulation of secondary bile acids and oxysterols. Oxysterol-CXC chemokine receptor 2 (CXCR2) axis plays a key role in the recruitment of tumor-promoting CD11b^+^Gr-1^+^ myeloid cells, and flow cytometry revealed an increase in the number of CXCR2 Mo-MDSCs, which in turn enhances innate immunosuppression and tumor progression 91.

Therapeutically, targeting LXR in MDSCs presents a viable strategy to modulate their immunosuppressive function. For instance, the dual-pH-sensitivity conjugated micelle system (PAH/RGX-104@PDM/PTX) effectively delivers LXR agonist RGX-104 and paclitaxel (PTX) to TME, enabling the coinstantaneous release of RGX-104 in perivascular regions to activate LXR in leukocytes, endothelial cells, and macrophages, thereby reducing MDSC levels and enhancing cytotoxic T lymphocyte infiltration 92.

### Arachidonic acid metabolism

Arachidonic acid metabolism involves the conversion of arachidonic acid, a polyunsaturated fatty acid, into various bioactive lipid mediators. This process begins with the release of arachidonic acid from membrane phospholipids by phospholipase A2. Arachidonic acid is then metabolized through three primary pathways: the cyclooxygenase (COX) pathway, the lipoxygenase (LOX) pathway, and the cytochrome P450 (CYP) pathway. The COX pathway produces prostaglandins and thromboxanes, the LOX pathway generates leukotrienes and hydroxyeicosatetraenoic acids (HETEs), and the CYP pathway forms epoxyeicosatrienoic acids (EETs) and additional HETEs.

For arachidonic acid metabolism in MDSCs, the roles of Prostaglandin E2 (PGE2), Cyclooxygenase 2 (COX2), and Fatty Acid Transport Protein 2 (FATP2) are particularly significant (**Figure 3**). PGE2 is a potent immunosuppressive mediator that enhances the immunosuppressive functions of MDSCs by inhibiting T cell and NK cell activities, thereby promoting tumor immune evasion 93. COX2 is an enzyme that converts arachidonic acid into prostaglandins, including PGE2. FATP2 facilitates the uptake of arachidonic acid into cells, ensuring a sufficient supply for eicosanoid synthesis, including PGE2 94. By targeting COX2 to inhibit prostaglandin synthesis, FATP2 to reduce arachidonic acid uptake, and PGE2 to block its immunosuppressive effects, it is possible to modulate arachidonic acid metabolism in a way that diminishes MDSCs' immunosuppressive functions.

### PGE2

In ovarian cancer, MDSC-derived PGE2 increases cancer stem-like cell properties and PD-L1 expression through the mammalian target of rapamycin (mTOR) pathway. Co-culture experiments indicate that PGE2 derived from MDSCs significantly enhances tumor PD-L1 expression and stemness, suggesting that depleting MDSCs might be therapeutically effective 95. PGE2 exerts its biological effects by binding to four different G protein-coupled receptors (EP1, EP2, EP3, EP4). EP2 and EP4 are two receptors that can regulate MDSC activity. They mainly activate adenylate cyclase through the G protein Gs subtype, leading to increased cAMP levels. As a second messenger, cAMP can activate protein kinase A (PKA) and cAMP response element binding protein (CREB), thereby promoting tumor cell proliferation and angiogenesis.

Tumor-derived PGE2 induces the nuclear accumulation of p50 NF-κB in M-MDSCs, diverting their response to IFN-γ towards immunosuppression by upregulating NO production and reducing TNF-α expression. Using PGE2 receptor antagonists EP2 (AH6809) and EP4 (AH23848) to block PGE2 receptors EP2 and EP4 can inhibit the nuclear accumulation of p50, reprogram M-MDSCs to a less suppressive phenotype, restore the anti-tumor activity of IFN-γ, and enhance the efficacy of cancer immunotherapy 96. The use of specific EP4 receptor inhibitors, such as MF-766, shows promise in enhancing anti-tumor immunity. EP4 inhibition by MF-766 synergistically improves the efficacy of anti-PD-1 therapy by modulating myeloid cells, NK cells, cDCs, and T cells, demonstrating the potential of combining EP4 antagonists with immune checkpoint inhibitors to enhance antitumor activity 97. Combination of the EP4 receptor antagonist ONO-AE3-208 with anti-PD-1 therapies promotes NK cell activation and shifts macrophages to a pro-inflammatory phenotype, which reduces hypoxia and normalizes tumor vasculature, thereby reducing tumor growth 98.

### COX2

By producing prostaglandins, including PGE2, COX2 regulates the immunosuppressive function of MDSCs. For instance, TFF3 can activate PMN-MDSCs *via* the NF-κB/COX2 pathway, enhancing their immunosuppressive capabilities by upregulating PGE2 production 99. Long noncoding RNAs (lncRNAs) such as lnc57Rik and Lnc-17Rik also modulate COX2 expression. Lnc57Rik upregulates genes associated with MDSC-mediated immunosuppression, including COX2, through interactions with C/EBPβ and histone modification enzymes, thereby enhancing PGE2 production and MDSC function in TME 100. Similarly, Lnc-17Rik facilitates the expression of COX2 by interacting with transcription factors and promoting histone modifications, leading to increased immunosuppressive activity of MDSCs and supporting tumor growth 101.

Studies have demonstrated that targeting COX2 can decrease MDSC accumulation and enhance anti-tumor immune responses. Celecoxib is an approved COX2 inhibitor in ovarian cancer and celecoxib-mediated COX2 blockade was observed to reverse MDSC suppressive function in an *in vitro* patient-derived culture assay. Synergistic effects of IFN-γ and TNF-α also lead to enhanced immunosuppressive activity of MDSC by inducing COX2 expression. MDSC overactivation and subsequent IDO, overexpression of iNOS, IL-10, and additional COX2 leads to a strong feedback suppression of type 1 immune responses. Upon blockade of COX2, this negative feedback mechanism would be broken, attenuating the inhibitory effect of MDSC on type 1 immune responses and thus promoting stronger anti-tumor immune responses 102. Furthermore, sulforaphane (SFN) from cruciferous plants effectively blocks PGE2 synthesis in breast cancer cells by activating Nrf2. This activation reduces COX2 expression and PGE2 secretion, prompting MDSCs to adopt an immunogenic phenotype and enhancing CD8^+^ T cell anti-tumor activities. Combining SFN with doxorubicin shows a significant decrease in tumor volume and MDSC expansion, highlighting SFN's potential as an adjuvant chemotherapeutic candidate 103. Shenqi Fuzheng Injection (SFI) has also been shown to inhibit the arachidonic acid metabolism process in melanoma cells, reducing the expression of COX2 and subsequently lowering PGE2 production. This inhibition leads to a decrease in MDSC and Treg levels while enhancing CD8^+^ and CD4^+^ T cell infiltration in TME. By improving the immune microenvironment, SFI enhances the anti-tumor effects of immune checkpoint inhibitors like the PD-L1 antibody 104. Moreover, Dectin-1, a receptor for β-glucans, is preferentially expressed on MDSCs. In a mouse model, the absence of Dectin-1 (Clec7a^-/-^) led to a reduction in MDSC-derived PGE2 levels and an increase in IL-22 binding protein expression, which is normally suppressed by PGE2. Dectin-1 signaling upregulates PGE2 synthases (such as PTGES2, PTGES3, COX1, and COX2), and administration of Dectin-1 antagonists (such as short-chained β-glucan laminin) inhibits intestinal tumorigenesis. This highlights the potential of targeting Dectin-1 to modulate PGE2 levels and MDSC function in colorectal cancer 105. To be note, there are many other clinically approved COX2 inhibitors, including Meloxicam and Etodolac, but their potential in targeting MDSCs have not yet been evaluated. Repurposing of these drugs may have promising prospects in the treatment of cancers.

### FATP2

FATP2 plays a pivotal role in regulating arachidonic acid metabolism in MDSCs. In addition to facilitating arachidonic acid uptake, a feedback loop exists between FATP2 and the RIPK3 pathway that drives COX2 expression and finally PGE2 production. Interestingly, the lack of RIPK3 in MDSCs induces NF-κB-dependent transcription of COX2. While PGE2 inhibited RIPK3 expression and promoted FATP2 expression, further amplifying the immunosuppressive function of PMN-MDSCs 106, 107. Targeting FATP2 to modulate arachidonic acid metabolism in MDSCs involves several strategies. Bladder cancer-derived exosomal circRNA_0013936 regulates FATP2 expression in PMN-MDSCs through the circRNA_0013936/miR-320a/JAK2 and circRNA_0013936/miR-301b/CREB1 pathways. This regulatory mechanism enhances the immunosuppressive activity of PMN-MDSCs by upregulating FATP2 and downregulating RIPK3, contributing to the suppression of CD8^+^ T cell functions 107. During fatty liver graft injury, arachidonic acid activates the NLRP3 inflammasome in MDSCs through FATP2, leading to increased IL-17 secretion by CD4^+^ T cells and promoting tumor recurrence post-transplantation 108. An effective approach is using pharmaceutical inhibitors like lipofermata, which block FATP2 activity. Inhibiting FATP2 reduces lipid accumulation and ROS production in MDSCs, thereby diminishing their immunosuppressive activity 109. Lipofermata also enhances the efficacy of PD-L1 blockade therapy by increasing the number of CD4^+^ and CD8^+^ T cells, as well as boosting CD107a expression on both CD4^+^ and CD8^+^ T cells 110. Lipofermata is the most representative FATP2 inhibitor known, and more FATP2 inhibitors may be developed in the future for cancer treatment and immunomodulation as research progresses.

## Amino acid metabolism

### Arginine metabolism

**Figure 4:**
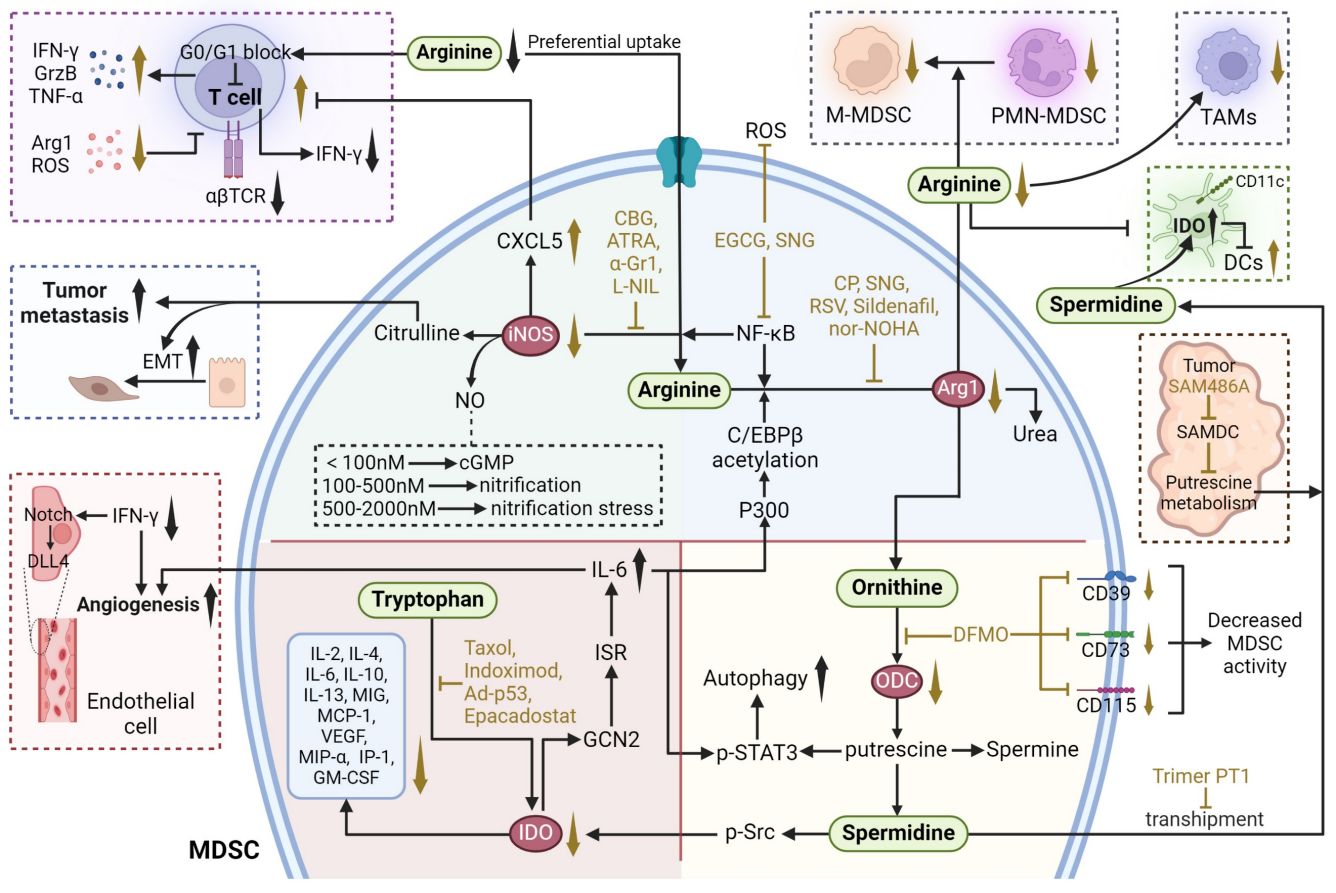
** Therapeutic strategies targeting amino metabolism in MDSCs.** ATRA all-trans retinoic acid, CBG cannabigerol, CP cyclophosphamide, DFMO difluoromethylornithine, DLL4 delta-like ligand 4, EGCG epigallocatechin-3-gallate, EMT epithelial-mesenchymal transition, GCN2 general control nonderepressible 2, ISR interferon-stimulated response element, L-NIL L-N6-(1-iminoethyl)-L-lysine, RSV resveratrol, SNG sanguinarine, Trp tryptophan. The black arrows show endogenous changes and the yellow arrows represent effects of potential therapies.

Arginine metabolism involves the conversion of the amino acid arginine into a variety of biologically active compounds, including NO, ornithine, polyamines, and citrulline. This process is regulated by key enzymes such as arginase and nitric oxide synthase (NOS). Arginase converts arginine to ornithine and urea, while NOS converts arginine to NO and citrulline (**Figure 4**).

### ARG1

ARG1 is a cytosolic protein that catalyzes the hydrolysis of arginine to ornithine and urea 111. In humans, ARG1 is mainly expressed by hepatocytes and cells of myeloid lineage. ARG1 has been identified as a pivotal immunosuppressive target, a sufficient amount of extracellular arginine is always necessary for the proliferation and cytotoxic function of T cells. When arginase hydrolyzes and depleted extracellular arginine, the expression of T cell receptor chain is inhibited and T cells are arrested in the G0-G1 phase, whereas arginine supplementation reverses this phenomenon and facilitates T cell expansion and anti-tumoral activities 112, 113. In addition, some of the environmental conditions such as acidosis, hypoxia and changes of ECM proteins can also induce ARG1 expression through hypoxia-inducible factors, lactic acid and other targets, as discussed above 111, 114. The overexpression of ARG1 in MDSCs is also modulated by P300-dependent acetylation of the transcription factor C/EBPβ 115.

Two major classes of arginase inhibitors have been evaluated in various cancers: synthetic and natural compounds. Chemical arginase inhibitors include NOHA and its analogs, 2(S)-amino-6-boronohexanoic acid, S-(2-boronylethyl)-L-cysteine, L-norvaline, and CB-1158, while natural arginase inhibitors include picrotoxin, chlorogenic acid (CA), Sanguinarine (SNG), and Resveratrol (RSV) 116. Both natural compounds and pharmacological agents targeting ARG1 in MDSCs have shown potential in cancer treatment. SNG are now recognized as potential treatments for lung cancer. SNG reduced the proportion of MDSCs and also increased the production of T helper 1 (Th1), T helper 2 (Th2), CTLs, macrophages, and DCs. *In vitro* studies revealed that SNG induced differentiation of MDSCs into macrophages and DCs *via* the NF-κB pathway, thereby down-regulating the proportion of MDSCs and promoting their apoptosis, differentiation, and maturation. Meanwhile, SNG also decreased the expression of ARG1, iNOS and eliminated ROS in MDSCs, and reduced the inhibitory effect on CD8^+^ T cell proliferation 117. RSV is an AhR antagonist that reduces the number of MDSCs, decreases the expression of ARG1 in MDSCs, and attenuates the immunosuppressive function of MDSCs. At the same time, RSV was able to increase F4/80^+^ macrophages and decrease CD11C^+^ DCs 118.

An increase in the proportion of MDSCs has also been reported after the administration of chemotherapeutic agents such as cyclophosphamide (CP) and docetaxel. The increase in the proportion of MDSCs after chemotherapy may attenuate anticancer T cell responses by decreasing L-arginine and L-tryptophan levels in the hormonal host. After combination therapy with oral supplementation of L-arginine, although there was no significant change in the proportion of MDSCs, there was a significant increase in the proportion of CD8^+^ T cells, and the antitumor effect of CP was enhanced 119. Furthermore, combinational therapies targeting ARG1 have shown promising applications. Radiation therapy (RT) has been found to recruit MDSCs to TME, where they suppress immune responses by elevating ARG1 expression. Administration of ARG1 inhibitor nor-NOHA and phosphodiesterase 5 (PDE5) inhibitor Sildenafil after RT reduced ARG1 expression and MDSC accumulation, enhanced infiltration of CD8^+^ T cells in the tumor, significantly elevated IFN-γ secretion by CD8^+^ T cells and delayed tumor regeneration after irradiation 120.

### iNOS

iNOS catalyzes the conversion of L-arginine to NO and citrulline. Multiple relationships exist between NO and tumor: appropriate concentration of NO promotes tumor growth, while high concentration of NO is detrimental to tumor growth and has anti-tumor effects. Antitumor activity mediated by NO levels can be divided into three main categories: cGMP signaling (<100 nM NO), pro-oncogenic nitrosative signaling (100-500 nM NO), and nitrosative stress signaling (500-2000 nM NO) 121. Meanwhile, citrulline can regulate apoptosis and differentiation to promote EMT and tumor metastasis 122.

The role of iNOS in MDSC-mediated immunosuppression is evident in various cancer models. For instance, in a squamous cell carcinoma (SCC) model, inhibition of iNOS with L-N6-(1-iminoethyl)-L-lysine (L-NIL) reduced lung metastasis by decreasing tumor-infiltrating myeloid cells and plasma Cxcl5 levels while promoting T-cell activation 123. Similarly, in hepatocellular carcinoma, all-trans retinoic acid (ATRA) reduces the expression of immunosuppressive molecules such as iNOS and the number of MDSCs, thereby shifting TME towards an anti-tumor phenotype and increasing cytotoxic T-cell infiltration 124. Treatment with epigallocatechin-3-gallate (EGCG), a compound derived from tea leaves, significantly inhibits MDSC accumulation and induces their apoptosis. EGCG down-regulates the canonical pathways in MDSCs, including the ARG1/iNOS/Nox2/NF-κB/STAT3 signaling pathway, thus ameliorating immunosuppression and enhancing T-cell responses 125. Additionally, cannabinoid-based therapies, such as cannabigerol (CBG), have also been found to reduce iNOS expression in MDSCs, restoring CD8^+^ T-cell activation 126. Moreover, recent studies have shown that anti-programmed cell death ligand 1 (αPD-L1) alone is insufficient in many cases due to the presence of immunosuppressive myeloid cells that reduce its efficacy. The infiltration of CTLs within the tumor, as well as the expression of GrzB and TNF-α in these CTLs, was significantly enhanced when CBG was combined with αPD-L1 therapy 126.

### Tryptophan metabolism

Tryptophan metabolism involves the breakdown of the amino acid tryptophan into several metabolites, including kynurenine, serotonin, and melatonin. As an essential metabolic enzyme in pathogenic inflammatory processes, IDO transforms tryptophan into a series of toxic downstream kynurenine metabolites (**Figure 4**). IDO has been found highly expressed in various kinds of human cancer cells and antigen-presenting cells, which is mainly induced by IFN-γ secreted from tumor-infiltrating lymphocytes through the activation of STAT1 [28656203]. Increased expression of IDO in tumor cells have further been identified to enhance the recruitment and activation of MDSCs. Rikke and colleagues have established a B16 melanoma model overexpressing IDO (B16-IDO) and found that MDSCs sorted from B16-IDO tumors showed significantly higher ARG1 expression, increased NO production, and upregulated TNF-α, IL- 10, IL-4, IL-6, IL-2, IFN-γ, IL-13, MIG, MCP-1, MIP-1α, IP-10, VEGF, and GM-CSF that related to MDSC recruitment, amplification, activation, and function. Moreover, the combined use of ARG1 inhibitors with iNOS inhibitors completely blocked the inhibitory activity of B16-IDO-derived MDSCs, emphasizing the potential interplay between tryptophan and arginine metabolism, but the exact mechanisms remain to be further investigated 127. In chronic lymphocytic leukemia (CLL), the accumulation of IDO-expressing monocytic MDSCs facilitates immune suppression. Research utilizing the Eµ-TCL1 mouse model of CLL demonstrated that the upregulation of IDO in MDSCs leads to an elevated kynurenine to tryptophan ratio in the serum, correlating with enhanced immunosuppression and progression of leukemia 128. Moreover, the pro-cancer role of IDO extends beyond immunosuppression to include promoting tumor angiogenesis. IDO expression in a subset of CD11^blo^ Gr-1^+^ MDSCs, termed IDVCs (IDO-dependent vascularizing cells), drives neovascularization by interfacing with inflammatory cytokines like IFN-γ and IL6. IDO induction in these cells provides a negative-feedback mechanism. Specifically, IDO1 activates and promotes IL-6 production by inducing the GCN2-ISR pathway. In turn, IL-6 facilitates neointima formation by preventing IFN-γ from exerting its antiangiogenic effects. This is achieved by neutralizing the impact of IFN-γ on the downregulation of delta-like ligand 4 (DLL4) within the Notch signaling pathway in endothelial cells 129.

Collectively, the interaction of IDO with tumor immunity has raised the possibility of targeting tryptophan catabolism to treat cancers. Several IDO inhibitors with reported activity on tumor immunity, such as Indoximod and Epacadostat, are currently being evaluated in clinical trials. Indoximod is the first identified competitive inhibitor and is the most employed IDO inhibitor, with a Ki value of 34 μM. Indoximod has been granted as orphan-drug by FDA for the treatment of melanoma at stage IIb to stage IV 130. However, in most clinical trials, Indoximod exhibited limited anti-tumor activity as a single agent, while combination of it with other therapies showed remarkable enhanced efficacy 131, 132. It has been found that Indoximod together with paclitaxel exhibited synergistic effects on autochthonous breast cancer, which was associated with a reduction in the expression of enzyme IDO from 12.06% to 3.56% 133. A randomized phase I/II trial (NCT01042535) involving patients with metastatic breast cancer indicated that the combination therapy of Indoximod and adenovirus-p53 transduced DC vaccine produced a chemosensitization effect. Furthermore, the maximum dose of Indoximod (1600 mg twice daily) was well tolerated 131. Additionally, the combination of Indoximod and temozolomide is also open now treating refractory primary malignant brain tumors of adult patients. Furthermore, Epacadostat is an orally available reversible competitive IDO inhibitor designed by Andrew, containing several underutilized functional groups form Indoximod including a hydroxyamidine, bromide, furazan, and sulfamide. Epacadostat exhibited potent inhibiting activity on IDO with a IC_50_ of 73 nM 134. Recently, the treatment of Epacadostat on a bilateral LLC tumor model of mice significantly reduced the number of MDSCs in the tumor 135. Clinically, the combinations of Epacadostat with immune checkpoint blockade therapies realized a synergistic effect (NCT03414229, NCT02364076, NCT03291054, NCT01604889, NCT02327078).

### Polyamine Metabolism

The polyamines putrescine, spermidine, and spermine are important metabolites of arginine and l-ornithine (**Figure 4**). In cancer, dysregulation of polyamine metabolism is frequently observed, and elevated levels of polyamines have been shown to be essential for tumor progression. However, the study of the mechanism of action between polyamines and TME is emerging. Putrescine is the first polyamine in the polyamine synthesis pathway and a precursor for the synthesis of spermidine and spermine 136. Ornithine can be further metabolized into putrescine, a polyamine that, in turn, can be transformed into spermidine and then spermine. Ornithine decarboxylase (ODC) is the rate-limiting enzyme of this process. In a model of glioblastoma, putrescine promotes immunosuppressive phenotypic differentiation of MDSCs through activation of STAT3 and enhances the survival of MDSCs by inducing their autophagy 137. Additionally, in DCs, spermidine produced by ODC substitute for TGF-β to activate Src kinase, which phosphorylates IDO and triggers immunosuppressive IDO signaling. IDO metabolizes tryptophan into immunosuppressive kynurenine, thereby contributing to the immunosuppressive phenotypic differentiation of MDSCs. MDSCs further export spermidine into the TME, thereby supplying DCs with additional polyamines to exacerbate IDO expression 138.

α-Difluoromethylornithine (DFMO) is an FDA-approved drug that specifically inhibits ODC 137. In melanoma-loaded mice, DFMO treatment significantly decreased the expression and activity of arginase in MDSCs and reduced arginine metabolism, resulting in decreased immunosuppression. DFMO also decreased the expression of CD39, CD73, and CD115 in MDSCs, inhibited CD39/CD73-mediated adenosine metabolism, and reduced the production of ATP, attenuating their immunosuppression 139. A novel combination therapy (polyamine blocking therapy, PBT) that blocks both the production and transport of polyamine by co-treatment of DFMO with a trimeric polyamine transport inhibitor (Trimer PTI) has been proposed. By decreasing their protective autophagy, PBT reduced the number of MDSCs and thus reversing the immunosuppressive TME. Combining PBT with αPD-1 exerts synergistic antitumor effects, significantly inhibiting 4T1 tumor growth and improving survival 137.

There are several other inhibitors targeting polyamines. S-adenosylmethionine decarboxylase (SAMDC) is a key enzyme in the synthetic pathway of spermidine and spermine. SAM486A is a specific SAMDC inhibitor that reduces intracellular polyamine levels in tumor cells by blocking the conversion of putrescine to spermidine and spermine 140, 141. Trimer44NMe is an inhibitor of polyamine transport, affecting cell proliferation and apoptosis by inhibiting the transmembrane transport of polyamines and reducing their intracellular uptake 142, 143. However, the studies of the above polyamine inhibitors are still mainly focused on direct anti-tumor effects, and it is still unclear about their role in targeting and regulating MDSCs. Since the metabolic reprogramming mechanism of MDSCs is similar to that of tumor cells, targeting polyamine metabolism in MDSCs may be potential therapies for reversing the development of immunosuppressive TME, which warrants further studies.

## Adenosine Metabolism

**Figure 5:**
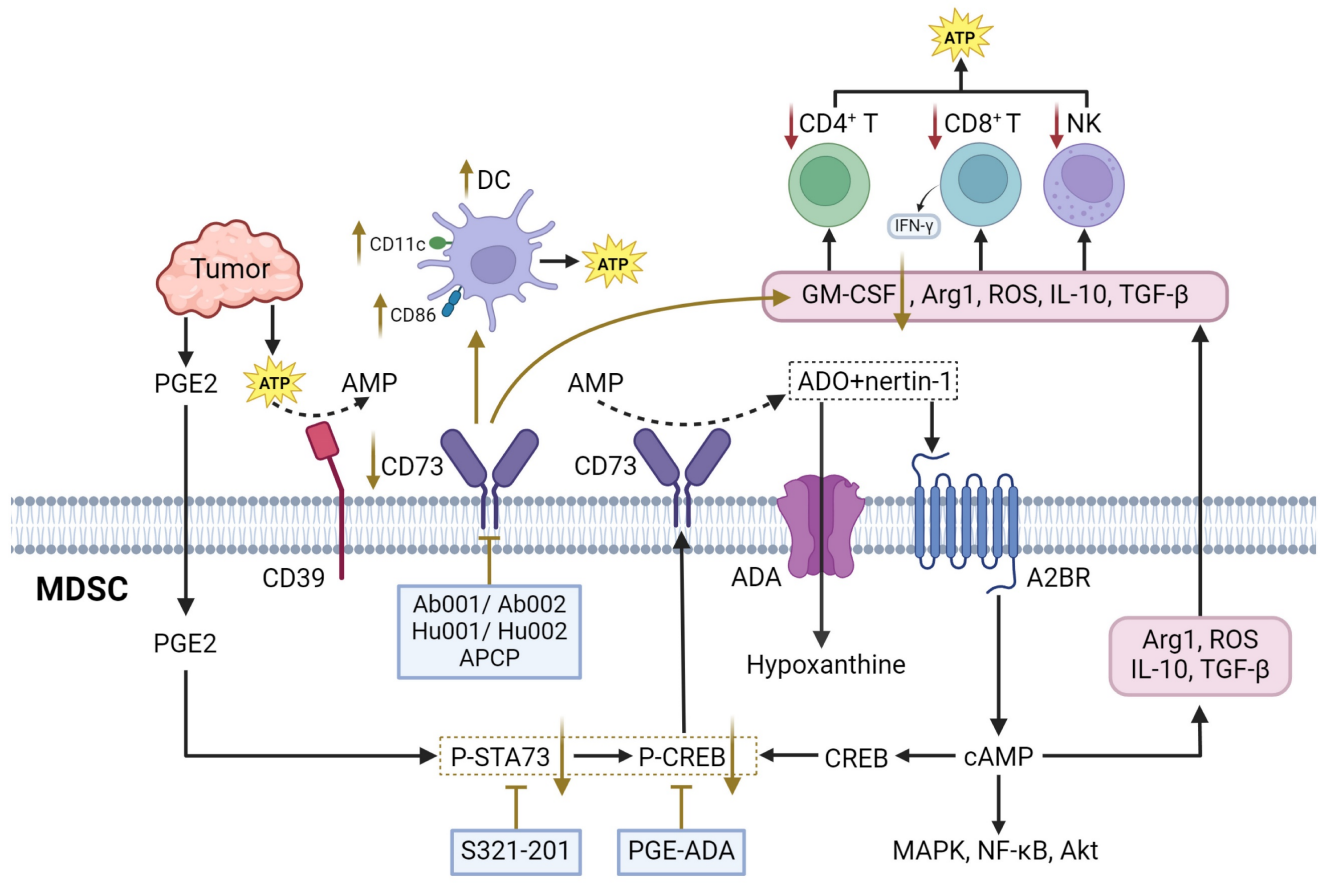
** Therapeutic strategies targeting adenosine metabolism in MDSCs.** A2BR adenosine A2B receptor, ADA adenosine deaminase, AMP adenosine monophosphate, APCP Adenosine 5′- (α, β-Methylene) diphosphate, ATP adenosine triphosphate, cAMP cyclic adenosine monophosphate, CREB cAMP response element binding protein. The black arrows show endogenous changes and the yellow arrows represent effects of potential therapies.

Adenosine is an evolutionary ancient regulator of metabolism that links energy states to physiological processes and plays a central role in a variety of physiological processes. Adenosine is generated mainly through the degradation of extracellular ATP and the release of intracellular metabolites. In TME, tumor cells and immune cells release ATP in large quantities into the extracellular space as a result of hypoxia, inflammation, or cellular damage. CD39 progressively degrades ATP to AMP, which is subsequently converted to adenosine by CD73. CD39 and CD73 are commonly upregulated in MDSCs and TME, leading to elevated adenosine levels, which enhances MDSC-mediated immunosuppression 144. Besides, adenosine can also be generated *via* intracellular metabolic pathway and released extracellularly *via* nucleoside transporters. Both of these metabolic pathways work in coordination to maintain high levels of adenosine in TME. Extracellular adenosine then activates downstream signaling pathways by binding to A2A and A2B adenosine receptors on the surface of MDSCs, leading to elevated intracellular cAMP levels (**Figure 5**). This signaling process enhanced the immunosuppressive function of MDSCs and inhibited the activity of anti-tumor immune effector cells such as T cells and NK cells 145. Furthermore, MDSCs express adenosine Deaminase, an enzyme that converts adenosine to hypoxanthine, reducing the local concentration of adenosine. However, the rate of adenosine production in MDSCs is usually higher than its clearance rate. This metabolic imbalance favoring adenosine signlaing ensures that MDSCs remain in a constant state of immunosuppression, thereby promoting tumor growth and progression 146.

### CD39 and CD73

CD39, an enzyme known as exonucleoside triphosphate diphosphate hydrolase-1 (ENTPD1), regulates adenosine metabolism in MDSC by hydrolyzing extracellular ATP and ADP to AMP, whereas CD73, an ecto-5'-nucleotidase, converts extracellular AMP to adenosine 147. In ovarian cancer, MDSC with high expression of CD39 can promote tumor cell proliferation and cisplatin resistance. In addition, CD39-upregulated MDSC could promote the hydrolysis of immunogenic ATP to immunosuppressive adenosine 148. In pancreatic ductal adenocarcinoma (PDAC) models, reduced CD73 expression leads to decreased MDSC populations and lower adenosine levels, thereby enhancing anti-tumor immune responses by increasing IFN-γ production by CD4^+^ and CD8^+^ T cells 149. Similarly, in head and neck squamous cell carcinoma and non-small cell lung cancer, CD73 expression is associated with advanced clinical stages and immune suppression, further supporting its role in promoting tumor progression through adenosine generation 150, 151.

There are multiple therapeutic strategies that target CD39 and CD73 in MDSCs. One approach involves the use of anti-CD73 antibodies Ab001/Ab002 or Hu001/Hu002. This approach not only diminishes the immunosuppressive effects of MDSCs but also promotes the activation of effector T cells and DCs, improving the overall anti-tumor immune response 151. Another strategy is the use of ectonucleotidase inhibitors, such as adenosine 5'-(α, β-methylene) diphosphate (APCP), which can reduce the suppressive function of CD73^+^ MDSCs 150. Additionally, the expression of CD39 and CD73 on MDSCs is positively correlated with the activation of STAT3, which enhances the differentiation of monocytes into MDSCs. In oral squamous cell carcinoma (OSCC), blocking STAT3 with inhibitors such as S3I-201 has been shown to reduce the expression of CD39 and CD73, decrease adenosine synthesis, and inhibit the occurrence of MDSCs 152. Furthermore, targeting other signaling pathways that regulate CD73 expression, such as the CREB pathway activated by tumor-derived factors like PGE2, provides another therapeutic avenue to inhibit the immunosuppressive functions of MDSCs 146.

### A2BR

A2BR is a G protein-coupled receptor which mediates the physiological effects of adenosine through second messenger cAMP 144. Mechanistically, A2BR is significantly expressed in MDSCs, where the binding of adenosine to this receptor either inhibits or stimulates adenylate cyclase activity, leading to alterations in intracellular cyclic AMP (cAMP) levels. This, in turn, triggers downstream cAMP-dependent signaling pathways such as MAPK, CREB, and NF-κB 153. Activation of A2BR by adenosine or its ligands (netrin-1) activates the cAMP/protein kinase A signaling pathway, resulting in an increase in immunosuppressive molecules such as ARG1, ROS, IL10, and TGFβ, thereby enhancing the suppressive function of MDSCs. This pathway also promotes the phosphorylation of CREB in MDSCs. Activated CREB, in conjunction with STAT3, regulates CD73 expression in M-MDSCs, leading to elevated extracellular adenosine production, forming a positive feedback loop. This, in turn, inhibits the activation of CD8^+^ T-cells within tumors, fostering an immunosuppressive TME that supports tumor growth 146.

At present, receptor antagonists targeting A2BR are also being progressively used. PSB-1115 is a selective A2BR antagonist. Blockade of A2BR activity with PSB1115 or knockdown of A2BR with siRNA in MDSCs reversed the effects of netrin-1 on MDSC-mediated T cell suppression. In addition, ARG1 activity and ROS production were decreased by A2BR blockade 153. There are many other selective antagonists of A2BR, like MRS1754, GS-6201, ATL-801, primarily target conditions like myocardial infarction and ischemia/reperfusion (IR) injury. However, their effects on MDSCs remain inadequately characterized 154-156. A2BR is also highly expressed in many tumor cells and plays a direct role in regulating tumor growth, angiogenesis, and metastasis. By targeting A2BR, multiple components of TME can be simultaneously influenced, which renders A2BR antagonists particularly appealing for antitumor therapy, beyond their role in regulating MDSCs.

## Discussion

TME is characterized by harsh conditions such as high lactate levels, unfavorable pH, hypoxia and high ROS levels, which ultimately lead to cancer progression and immune escape. Furthermore, nutrients are limited in TME and the accumulation of metabolic wastes due to abnormal tumor metabolism exacerbates the hostile environment. TME includes multiple types of immune cells that support or suppress tumorigenesis and progression. Nutritional depletion and overproduction of metabolic byproducts driven by tumor development affect the metabolism of immune cells, thus hindering antitumor immunity.

The metabolic pattern of tumor-infiltrating immune cells in the TME is quite different from that of resting immune cells, and they undergo a "metabolic reprogramming" phenomenon to proliferate, differentiate, and perform effector functions. Briefly, intratumoral T cells can differentiate into various subtypes, such as effector T cells, memory T cells, and Treg, each with different metabolic characteristics. 157, 158. Memory T cells, like naïve T cells, rely on OXPHOS for ATP production and have a low glycolysis rate 159, 160. Activated T cells utilize OXPHOS to metabolize glucose for nucleotide and serine biosynthesis, enhancing L-arginine metabolism and inhibiting glycolysis 113, 161. Tregs primarily depend on FAO and OXPHOS for energy 162. NK cells in the TME are responsible for recognizing and killing tumor cells and primarily depend on glucose for energy. Studies have shown that excess fatty acids are harmful to NK cells 163, while activated NK cells maintain their function through glycolysis under hypoxic conditions 164. DCs are key antigen-presenting cells that rely on OXPHOS in their resting state but shift to aerobic glycolysis upon activation, with an increased demand for amino acids, especially glutamine 165-168. TAMs include M1 and M2 macrophages; M1 macrophages kill tumors through glycolysis and oxidative stress 169, while M2 macrophages rely on FAO and TCA to promote tumor growth 170, 171.

Metabolic reprograming is more complicated in MDSCs as multiple metabolic pathways crosstalk to each other under different circumstances. In the hypoxic TME, activation of HIF-1α induces a metabolic shift from OXPHOS to glycolysis in MDSCs 172. While CD36 upregulation-mediated lipid uptake could promote a shift from glycolysis to FAO in MDSCs. An abundant supply of lipids can enhance the metabolism of PUFA-related arachidonic acid and promote the production of PGE2. Thus, a lipid-rich TME, coupled with a robust lipid metabolism profile, would significantly facilitates the infiltration, survival, and expansion of MDSCs. As summarized in previous sections, it is noteworthy that the highly activated glycolytic pathway can also induce the reprogramming of amino acid metabolism in MDSCs, leading to a preference for depleting arginine and tryptophan to promote the generation and maintenance of an immunosuppressive TME. Moreover, studies suggest that MDSCs may exhibit enhanced uptake of glutamine, although conflicting *in vivo* and *in vitro* evidence exists 173. In essence, MDSCs tend to deplete critical metabolic substrates necessary for T cell activation and homeostasis, while releasing immunosuppressive metabolites, thereby participating in metabolic regulation of immune responses in the TME. This behavior closely resembles the metabolic dysregulation traits of tumor cells, which not only robs oxygen and nutrients but also limits nutrient supply and waste removal through abnormal angiogenesis.

From this perspective, the metabolic reprogramming undertaken by tumor cells and various immune cells within TME to sustain their respective functions results in distinct preferences for different nutrient sources. However, limited studies have systematically evaluated the competitive abilities of different cells within the TME for relevant nutrients or metabolites *in vivo*. Glucose uptake can be measured using [18F] fluorodeoxyglucose positron emission tomography imaging to detect cancers and monitor therapeutic responses. A previous study has shown that in a variety of mouse cancer models, it is not the tumor cells that consume the most glucose in the TME, but rather the myeloid-derived cells, followed by T cells 174. In contrast, tumor cells had the highest uptake capability of glutamine, and blocking glutamine uptake increased glucose uptake in all cell types. Although competitive glucose uptake under tumor microenvironmental conditions is responsible for impaired immune cell function, competitive uptake of other metabolites such as fatty acids, or growth factors by tumor cells and immune cells is equally important in influencing immune cell function 175. Studies have shown that MDSCs are primarily dependent on lipid and cholesterol metabolism, and the inhibition of lipid transport, lipolysis, and FAO has been shown to attenuate their immunosuppressive and pro-tumorigenic activities. However, which cell in the TME preferentially uptake lipids such as fatty acids has not been studied as intensively as glucose uptake studies. It may be possible to conduct in-depth studies with the help of isotope labeling and metabolomic analysis.

This limitation hampers our understanding of the heterogeneity of metabolic behaviors within tumors, potentially leading to unexpected outcomes when targeting specific pathways. For instance, targeting glycolysis-related pathways can effectively inhibit the energy metabolism of MDSCs and reduce their immunosuppressive capacity. However, this approach also affects the functional activity of other immune cells in the TME, such as NK cells, activated DCs, and M1-type TAMs, which are energy-dependent on glycolytic metabolism. The inhibition of the glycolytic pathway blocked the antigen-presenting function of DC cells, which led to the inability of T cells and NK cells to play a normal tumor-killing role. Similarly, although targeted inhibition of amino acid metabolism can reduce the release of immunosuppressive molecules such as ARG1, NO, and IDO1 in MDSCs and improve the tumor immunosuppressive microenvironment. However, T cells cannot exert their antitumor effects without the uptake of L-arginine and glutamine, and once the amino acid metabolic pathway is inhibited, the immune checkpoint inhibitor-mediated antitumor response is hindered 176. Hence, it is a complex challenge to precisely target metabolic pathways in MDSCs without damaging other immune cells or stromal cells. Therefore, leveraging advanced technological approaches, such as single-cell sequencing, single-cell metabolomics, and metabolic flux analysis, to investigate the utilization rates and forms of the same nutrient sources by different cells within the TME can aid in constructing a metabolic landscape that reflects cellular heterogeneity. Such endeavors will better facilitate the development of feasible intervention strategies aimed at simultaneously starving tumor cells, reversing the immunosuppressive phenotype of MDSCs, and enhancing the responses of tumor-killing cells.

To address the challenges associated with targeting the metabolism of MDSCs, it is crucial to recognize that metabolic pathways do not function in isolation. These pathways interact and regulate each other through common upstream signaling transduction, sharing intermediates, regulatory factors, and enzyme activities, forming a complex metabolic network. For instance, The PI3K/AKT/mTOR pathway is a frequently dysregulated pathway in various types of cancers that is critical for cell survival and growth under tumor pathological conditions and is one of the widely known crosstalk signals 177. Mediating the PI3K/AKT/mTOR pathway regulates glycolytic flux and ROS production in MDSCs 31. Activation of PI3K/AKT/mTOR enhances the activities of the key enzymes of glycolysis, HK2 and PKM2, and increases glycolytic flux in MDSCs 29. The downstream signaling of PI3K/AKT/mTOR, HIF-1α, is an important driver in maintaining glycolysis under hypoxic conditions in MDSCs and by inducing the expression of PD-L1, and releasing chemokines and inflammatory factors to shape the immunosuppressive microenvironment 20, 22, 95. In addition, PI3K/AKT/mTOR downstream signaling sterol regulatory element binding proteins (SREBPs) are key transcription factors that regulate lipid metabolism 178. SREBP1 enhances lipid metabolism through activation of adipogenic gene transcription, include FASN and SAD 179. Meanwhile, activated mTORC1 promotes SREBP2 maturation, which mediates cholesterol synthesis. And cholesterol accumulation can inhibit LXRβ-RXRα heterodimer by blocking LXRβ nuclear translocation, thus affecting arginine metabolism in MDSCs 90. However, targeting the PI3K/AKT/mTOR pathway can result in adverse events leading to early treatment or study cancellation as the PI3K/AKT/mTOR pathway plays a critical role in multiple cellular processes 180. By utilizing the multiple actions of a single target, we can more effectively coordinate the regulation of different metabolic pathways. Recognizing these interdependencies allows us to turn challenges into opportunities, ultimately improving the specificity of targeting MDSCs.

## Conclusion

Insights into metabolic reprogramming and immunosuppression in TME have highlighted the critical role of MDSCs in anti-tumor immunity. Current strategies for targeting MDSCs, either alone or in combination with immune checkpoint inhibitors and radiotherapy, show promise but also face significant challenges (**Table 1**). The need to selectively target MDSCs without adversely affecting other immune cells in the TME, such as memory CD8^+^T cells and DCs, is a major hurdle. In order to advance cell-targeted therapies, it is crucial to analyze the biological characteristics and metabolic profiles of MDSCs in different tumor types and stages using technologies such as single-cell sequencing and metabolomics. In summary, by addressing above challenges, targeting the rewired metabolic networks of MDSCs in the TME could provide a more precise approach to enhance tumor immunotherapy and to develope more effective and specific strategies for treating human malignancies.

## Figures and Tables

**Table 1:** Key experiments of interfering metabolism to regulate MDSC function and improve immune activity.

Metabolic pathway	Target	Drug	Impact
Glucose metabolism	mTOR	Rapamycin	The proportion of MDSCs (CD68^+^CD33^+^ and CD68^+^ARG1^+^) is reduced 30.
		Metformin	
		MENK	MDSC levels are significantly reduced by inhibiting IL-6, MCP-1, and TNF-α. Factors promoting MDSC production (G-CSF, GM-CSF, M-CSF, IL-6, IL-1β) and migration (KC, MCP-1, S100A8, S100A9, VEGF) are downregulated in the TME. T cell immunosuppression is inhibited by reducing iNOS, ARG1, IDO, PD-L1, HO-1, NOX2, and TGF-β in MDSCs 31.
		3PO	MDSC mature phenotypic differentiation is inhibited by effectively suppressing the expression of MHCII, CD80, and CD86, and glycolytic function is significantly reduced 27.
	STAT3	Napabucasin	MDSC apoptosis is increased. Pathways related to the cell cycle, mismatch repair, and DNA replication are significantly downregulated in association with reduced survival and proliferation, and pathways related to lysosomes, phagosomes, and antigen processing and presentation are significantly upregulated. The expression of IL-6, CD80, and CD86 is significantly upregulated, IL-1b and COX2 expression is significantly downregulated, whereas the expression of Ptges2, NOX2, ARG1, and iNOS and ROS production show a downregulation 36.
		JSI-124	The proportion of MDSCs (CD68^+^CD33^+^) is reduced 37.
		Dichloroacetate	The number of MDSCs (CD11b^+^Gr-1^+^) infiltrated is reduced 33.
		S3I-201	The differentiation of CD14^+^ monocytes to M-MDSC (CD14^+^HLA-DR^-/low^) is inhibited. Proliferation inhibition of T cells from MDSC is reduced by decreasing adenosine synthesis 152.
		Rg3-PTX-LPs	IL-6 expression is significantly reduced, and the number of MDSC (CD11b^+^Gr-1^+^) is strongly reduced 43.
	HK2	3-BrPA	The MDSC population is significantly reduced, and the infiltration of CD8^+^ T cell population in TME is increased 46.
	PKM2	MRIAN	The frequency of MDSCs in the BM and spleen is reduced, and the frequency of macrophages, DCs, NK cells, CD4^+^ T cells, CD8^+^ cells, and CD3^+^CD8^+^IFN-γ^+^ CTLs is recovered. ROS levels in MDSC are reduced 50.
		SHK@HA-MPDA	The lactate level of TME is decreased, the number and migration ability of MDSC are decreased, the number of NK cells and CD8 T cells infiltration is increased, and the expression of granzyme B and INF-γ is increased 51.
	LDH	R-mPDV/PDV/DOX/siL	G-CSF and GM-CSF cytokine production is significantly reduced, and MDSC (CD11b^+^Gr-1^+^) infiltration is significantly reduced in TME 57.
	MCT	AZD3965	Both MDSCs (CD14^+^HLA-DR^-^) and Tregs are significantly reduced 63.
Lipid metabolism	PPAR-γ	GW9662/ T0070907	The expression of ARG1 in MDSC is significantly inhibited, and the infiltration of CD8^+^ T cells is increased 76. PMN-MDSC immunosuppression and T cell exhaustion are diminished 77.
		9-HODE	The transendothelial migration capability of Ly6G^+^ MDSCs is reduced, ROS production is decreased, and aberrant mitochondrial membrane potential is improved 78.
		AZD1208	The abundance of CD11b^+^Gr-1^+^ MDSCs and M-MDSCs are significantly reduced, and PMN-MDSCs and CD11b^+^F4/80^+^ macrophages are mildly increased. IFN-γ^+^CD8^+^ T cells are significantly increased 79.
	CPT1A	Etomoxir	G-MDSCs (CD14^neg^CD33^+^HLA-DR^neg^CD66b^+^) are increased. Fatty acid uptake is increased 72.
	FASN	Ginger polysaccharide	Promoting apoptosis of MDSCs by inhibiting fatty acid synthesis and lipid droplet accumulation, and reducing the energy supply of cells 83.
	LXR	PAH/RGX-104@PDM/PTX	The abundance of MDSCs (CD11b^+^Gr-1^+^) are reduced 92.
	PGE2	AH6809, AH23848	The number of CD11b^+^Ly6C^+^ MDSCs and NOS2 expression are decreased, and TNF-α expression is increased while the number of tumor-infiltrating IFN-γ^+^CD8^+^ T cells is increased 96.
		MF-766	The inhibitory activity of MDSCs induced by GM-CSF/IL-4 is suppressed, and IFN release in response to TCR stimulation is increased, reversing the inhibition of T cell proliferation by MDSCs 97.
	COX2	Celecoxib	The expression of IDO, NOS2, IL10, IFN-γ and TNF-α are decreased 102.
		SFN	The expression of IL-10, ARG1, and TGF-β in MDSC is reduced, preventing the accumulation of CD11b^+^Gr-1^+^ MDSC, CD11b^+^Ly6C^+^ MDSCs, and CD11b^+^F4/80^+^ MDSCs 103.
		SFI	The infiltration of M-MDSCs and PMN-MDSCs in TME is significantly inhibited, and the ratio of CD8/CD4 T cells is significantly increased 104.
	FATP2	lipofermata	MitoSOX in MDSC is reduced, and the number of MDSC is significantly lower 108, 109.
Amino acid metabolism	ARG1	SNG	The expression of IL-6, IL-1β, IL-17A, S100A9, ROS, ARG1, and iNOS in MDSC is downregulated, reducing the abundance of MDSCs (CD11b^+^Gr-1^+^, CD11b^+^Ly6G^+^), inducing late apoptosis of MDSC. The percentage of Th1, Th2, and CTL is significantly increased 117.
		RSV	The number of MDSCs (CD11b^+^Ly-6G^+^Ly-6C^low^) is reduced, and the proliferation inhibition of T cells is blocked 118.
		Sildenafil	The accumulation of PMN-MDSCs (CD11b^+^ Ly-6G^+^ Ly-6C^low^) and ARG1 expression is reduced 120.
	iNOS	L-NIL	The total number of Ly6G^+^ myeloid cells infiltrated in the TME is reduced, and the expression of Icam1, MMP2, MMP3, and MMP9 is decreased 123.
		ATRA	Significantly lower levels of MDSCs (CD11b^+^ Ly6C^-^ Ly6G^+^) are observed, with significantly lower protein levels of Arg1, iNOS, IDO, S100A8 and S100A9, and significantly higher numbers of Granzyme B-positive immune cell in TME 124.
		EGCG	The percentages of MDSCs (CD45^+^ CD11b^+^ Gr-1^+^) are reduced, MDSC viability is inhibited, and MDSC apoptosis is increased 125.
	iNOS	CBG	The expansion of Ly6C^high^ MO-MDSCs is specifically affected. MDSCs express lower levels of iNOS, leading to the restoration of CD8^+^ T cell activation 126.
	IDO	Epacadostat	IDO expression is inhibited, and fewer tumor-infiltrating MDSCs are observed 135.
	ODC	DFMO	MDSCs (Gr-1^+^ CD11b^+^) has elevated ODC activity and significantly lower expression of CD39, CD73, CD115, and ARG1 139.
Adenosine metabolism	CD73	APCP	The inhibition of T cells by CD73^+^ PMN-MDSCs is attenuated, restoring IFN-γ expression 150.

MENK, Methionine enkephalin, 3PO, 3-(3-pyridinyl)-1-(4-pyridinyl)-2-propen-1-one, JSI-124, Cucurbitacin I, S3I-201, 2-Hydroxy-4-(((4-methylphenyl) sulfonyloxy) acetyl) amino)-benzoic acid, PTX, Paclitaxel, 3-BrPA, 3-Bromopyruvic acid, MRIAN, metabolically reprogrammed immunosurveillance-activated nanomedicine, GW9662/T0070907, 2-chloro-5-nitro-N-4-(3-trifluoromethylbenzyl) benzene sulfonamide, 9-HODE, 9-Hydroxy-10,12-octadecadienoic acid, MF-766, 4-[1-[(1-[4-(Trifluoromethyl) benzyl]-1H-indol-7-yl) carbonyl] amino] cyclobutanecarboxylic acid, SFN, Sulforaphane, SFI, Shenqi Fuzheng Injection, SNG, Sanguinarine, RSV, Resveratrol, L-NIL, L-N6-(1-iminoethyl)-L-lysine, ATRA, All-Trans Retinoic Acid, EGCG, Epigallocatechin-3-gallate, CBG, Cannabigerol, DFMO, α-Difluoromethylornithine, APCP, Adenosine 5'-(α, β-methylene) diphosphate.

## Abbreviations

ARG1: arginase 1
CAMKK2: calcium/calmodulin-dependent protein kinase kinase 2
CREB: cAMP response element binding protein
CPT1A: carnitine palmitoyltransferase 1A
CTLs: cytotoxic T lymphocytes
DCs: dendritic cells
ECM: extracellular matrix
FAO: fatty acid oxidation
FATP2: fatty acid transport protein 2
GLUT1: glucose transporter type 1
HK2: hexokinase 2
IDO: indoleamine 2,3-dioxygenase 1
iNOS: inducible nitric oxide synthase
LDHA: lactate dehydrogenase A
LXR: liver X receptor
LDL: low-density lipoproteins
MCT: monocarboxylate transporters
MDSCs: myeloid-derived suppressor cells
NK: natural killer
OXPHOS: oxidative phosphorylation
PPARγ: peroxisome proliferator-activated receptor γ
PUFA: polyunsaturated fatty acids
PGE2: prostaglandin E2
PKM2: pyruvate kinase M2
ROS: reactive oxygen species
TAMs: tumor-associated macrophage
TME: tumor microenvironment
